# A physics-informed multi-modal transformer framework for predictive maintenance and remaining useful life estimation of electrical submersible pumps

**DOI:** 10.1038/s41598-026-56666-4

**Published:** 2026-06-22

**Authors:** Yasin Khalili, Mohammad Ahmadi, Mostafa Keshavarz Moraveji

**Affiliations:** 1 Department of Petroleum and Geoenergy Engineering, Amirkabir University of Technology, Tehran, Iran; 2 Department of Chemical Engineering, Amirkabir University of Technology, Tehran, Iran

**Keywords:** Electrical Submersible Pumps (ESPs), Predictive Maintenance, Remaining Useful Life Estimation, Physics-Informed Machine Learning, Multi-Modal Transformer, Industrial Prognostics, Deep Learning, Prognostics and Health Management (PHM), Energy science and technology, Engineering, Mathematics and computing

## Abstract

Electrical Submersible Pumps (ESPs) are widely used in oil and gas production but are highly vulnerable to mechanical, electrical, hydraulic, and thermal degradation under harsh downhole conditions. Unexpected ESP failures can result in severe production losses, costly interventions, and reduced operational reliability, highlighting the importance of predictive maintenance and Remaining Useful Life (RUL) estimation. Existing prognostic approaches often rely on single-modality data and lack physical consistency in degradation modeling. This study proposes HF-ESPNet, a physics-informed multi-modal transformer framework for joint ESP failure prediction and RUL estimation. The proposed model integrates heterogeneous operational data, including electrical telemetry, pressure, temperature, vibration indicators, well metadata, and maintenance history within a unified transformer-based architecture. Domain-specific physics-informed constraints related to hydraulic behavior, thermal dynamics, and vibration degradation are incorporated into the learning objective to improve physical consistency and prognostic reliability. The framework was evaluated using an anonymized real-world ESP dataset containing more than 58,000 multivariate time-series samples under diverse operational conditions. Experimental results demonstrate that HF-ESPNet outperforms conventional machine learning, deep learning, and transformer-based baseline models in both failure prediction and RUL estimation tasks. Ablation analysis further confirms the importance of vibration-derived features and physics-informed constraints in improving predictive robustness and degradation modeling accuracy. The results demonstrate that combining multi-modal representation learning, transformer-based temporal modeling, and physics-informed learning provides a robust and interpretable framework for ESP prognostics, supporting improved maintenance scheduling, reduced unplanned downtime, and enhanced operational reliability in oil and gas production systems.

## Introduction

Electrical Submersible Pumps (ESPs) are among the most widely deployed artificial lift systems in upstream oil and gas production, particularly in wells where natural reservoir pressure is insufficient to sustain economic flow rates^1^. ESP systems operate under harsh downhole conditions characterized by high temperatures, fluctuating pressures, abrasive fluids, gas interference, and electrical loading variations. These conditions accelerate mechanical wear, thermal degradation, and electrical stress, frequently leading to premature failures^2^. Unplanned ESP shutdowns result in significant production losses, costly workover operations, and increased operational risk, making reliability and condition monitoring critical concerns for field operators^3^.

Traditionally, ESP maintenance strategies have relied on reactive or preventive approaches. Reactive maintenance responds only after catastrophic failure, often incurring substantial downtime and intervention costs^4^. Preventive maintenance, while reducing unexpected failures, is typically based on fixed schedules that do not reflect the actual health state of the pump and may lead to unnecessary interventions. These limitations have motivated a shift toward predictive maintenance strategies, which aim to anticipate failures before they occur by leveraging operational data and advanced analytics^5^.

With the increasing availability of high-frequency ESP monitoring data, data-driven methods based on machine learning and deep learning have gained considerable attention. Previous studies have explored a range of approaches, including statistical models, decision trees, random forests, convolutional neural networks, and recurrent neural networks, to detect abnormal operating conditions and predict failure events^6^. While these methods have demonstrated promising results, several limitations remain. Many existing approaches rely on a single data modality most commonly electrical or pressure telemetry thereby overlooking valuable information contained in vibration signals, thermal behavior, and operational context. Moreover, a large portion of the literature frames ESP prognostics as a binary classification problem, focusing solely on failure detection rather than continuous degradation modeling and Remaining Useful Life (RUL) estimation, which is more informative for maintenance planning^7^.

More recently, transformer-based architectures have been introduced for time-series forecasting due to their ability to model long-range temporal dependencies more effectively than recurrent networks^8^. Despite their success in various industrial applications, transformer-based models applied to ESP systems remain largely data-driven and statistical in nature^9^. They generally lack integration with physical knowledge of pump behavior, such as hydraulic affinity laws, thermal dynamics, and vibration growth patterns. As a result, these models may produce predictions that are difficult to interpret and may not generalize well across different wells and operating conditions^10^.

Another challenge in ESP predictive maintenance research is the limited accessibility of openly available field datasets. Due to confidentiality constraints in oil and gas operations, most ESP operational data cannot be publicly shared^11,12^. Consequently, many studies either omit detailed descriptions of data provenance or rely on simplified representations that limit reproducibility and industrial relevance. Addressing this issue requires carefully curated operational datasets that preserve real-world degradation characteristics while ensuring anonymization and compliance with data-sharing restrictions^13^.

Unlike existing ESP predictive maintenance approaches that rely on single-modality data or purely data-driven models, the proposed framework explicitly integrates domain-specific physical knowledge with multi-modal transformer-based learning. This combination enables physically consistent predictions while capturing complex degradation dynamics across multiple sensing modalities, thereby addressing key limitations of existing approaches.

To address the aforementioned limitations, this study proposes a physics-informed, multi-modal transformer-based framework for ESP predictive maintenance. The proposed approach integrates heterogeneous operational data including electrical telemetry, pressure and temperature measurements, vibration-derived indicators, well metadata, and maintenance records within a unified learning architecture. By embedding physics-informed constraints into the learning process, the model enforces physically consistent predictions and improves generalization across diverse operating conditions. Furthermore, the framework jointly performs failure prediction and RUL estimation, providing both early warning capabilities and actionable time-to-failure information for maintenance planning.

The key novelty of this work lies in the integration of domain-specific physics-informed constraints tailored to ESP systems, multi-modal data fusion within a transformer-based architecture, and a unified multi-task learning framework for simultaneous failure prediction and Remaining Useful Life estimation.

While the proposed framework incorporates several components, including multi-modal data fusion, transformer-based temporal modeling, and joint failure prediction and RUL estimation, the primary contribution of this work lies in the formulation and integration of domain-specific physics-informed constraints within the learning objective.

Specifically, the proposed approach introduces a structured physics-informed loss function derived from ESP operating principles, which constrains the model to produce physically consistent predictions while learning from data. The remaining components of the framework, including multi-modal feature fusion and multi-task learning, serve to support and enhance this core formulation by enabling richer representation learning and improved predictive performance.

The remainder of this paper is organized as follows. Section 2 reviews related work on ESP predictive maintenance, machine learning–based prognostics, and physics-informed modeling. Section 3 describes the proposed methodology, including dataset preparation, model architecture, and evaluation procedures. Section 4 presents experimental results and a detailed discussion of model performance, interpretability, and ablation studies. Finally, Sect. 5 concludes the paper and outlines directions for future research.

## Literature review

Predictive maintenance has emerged as a key research area in industrial systems due to its potential to reduce unplanned downtime, optimize maintenance scheduling, and extend equipment life^14^. In the oil and gas industry, ESPs represent a critical application domain for predictive maintenance because of their widespread use and high failure-related costs^15^. This section reviews prior work related to ESP condition monitoring, data-driven predictive maintenance approaches, deep learning–based prognostics, transformer-based time-series modeling, and physics-informed machine learning, with the objective of identifying existing limitations and research gaps.

### ESP failure mechanisms and condition monitoring

ESPs operate under harsh downhole conditions characterized by high temperature, pressure fluctuations, multiphase flow, and mechanical stress, which collectively accelerate system degradation and increase the likelihood of failure. Extensive studies have investigated ESP failure mechanisms from both mechanical and operational perspectives, identifying common failure modes such as bearing wear, shaft misalignment, insulation breakdown, gas lock, scale deposition, and thermal overload^16^. These failure modes arise from different physical processes and evolve over time, often exhibiting complex and nonlinear degradation behavior.

Comprehensive analyses have shown that these failure mechanisms manifest through observable changes in multiple sensing modalities, including electrical load, pressure behavior, temperature profiles, and vibration signatures^17^. For example, mechanical degradation processes such as bearing wear and shaft misalignment typically result in increased vibration amplitudes and characteristic frequency-domain patterns, accompanied by gradual temperature rise due to friction. Electrical faults, including insulation degradation, often manifest as current instability and abnormal thermal behavior. Hydraulic issues such as gas lock or scale deposition led to pressure fluctuations, reduced flow efficiency, and variations in motor load. Thermal overload conditions are reflected in sustained temperature increases and increased electrical demand. As a result, modern ESP monitoring systems are designed to collect multivariate telemetry data encompassing electrical, hydraulic, thermal, and mechanical measurements, which provide complementary information about system health^18^.

To explicitly illustrate the relationship between ESP failure mechanisms and their observable signatures, **Table **1 presents a mapping between common failure modes, underlying physical processes, and the corresponding measurable variables. This mapping highlights that different failure mechanisms are associated with distinct yet overlapping signatures across multiple sensing domains.

**Table 1:** Mapping of ESP Failure Modes to Observable Variables and Signatures.

Failure mode	Physical mechanism	Observable variables	Typical signatures
Bearing wear	Mechanical degradation due to friction and fatigue	Vibration, temperature	Increased vibration amplitude, rising temperature, frequency band energy growth
Shaft misalignment	Mechanical imbalance and misalignment	Vibration, current	Harmonic vibration patterns, load fluctuations
Insulation breakdown	Electrical degradation of motor insulation	Current, temperature	Current instability, abnormal heating, thermal drift
Gas lock	Gas interference in pump intake	Pressure, current	Pressure fluctuations, reduced pump efficiency, load instability
Scale deposition	Flow restriction due to solid accumulation	Pressure, temperature	Increased discharge pressure, reduced flow efficiency
Thermal overload	Excessive heat generation under load	Temperature, current	Sustained temperature rise, increased electrical load

This mapping demonstrates that ESP degradation is inherently a multi-modal process, in which no single variable can fully capture system health. Instead, reliable condition monitoring and prognostic modeling require the integration of heterogeneous data sources that reflect electrical, mechanical, hydraulic, and thermal behavior simultaneously. This multi-dimensional nature of degradation poses significant challenges for traditional monitoring approaches.

Early ESP condition monitoring systems primarily relied on rule-based thresholds and expert-driven interpretation of individual variables. While such approaches are simple and interpretable, they struggle to capture nonlinear interactions among variables and often generate false alarms under dynamic operating conditions^19^. In particular, threshold-based methods are unable to effectively distinguish between transient operational variations and genuine degradation patterns, limiting their reliability in complex environments.

These limitations have motivated the adoption of data-driven approaches capable of learning degradation patterns directly from historical operational data. However, many existing models still rely on single-modality inputs and do not fully exploit the complementary information available across different sensing domains. This gap highlights the need for advanced multi-modal modeling frameworks that can jointly analyze diverse data sources and capture the complex interactions underlying ESP degradation processes.

### Machine learning approaches for ESP predictive maintenance

With the increasing availability of ESP operational data, machine learning techniques such as decision trees, support vector machines, random forests, and k-nearest neighbors have been applied to failure detection and classification tasks^20^. These methods demonstrated improved performance over rule-based systems, particularly in identifying abnormal operating conditions. However, most traditional machine learning models require extensive feature engineering and are limited in their ability to model long-term temporal dependencies inherent in ESP degradation processes^21^.

To address these challenges, deep learning models including convolutional neural networks (CNNs) and recurrent neural networks (RNNs) have been introduced for ESP prognostics^22^. CNN-based approaches have been effective in extracting local temporal and spectral features from sensor signals, while Long Short-Term Memory (LSTM) networks have shown improved capability in modeling sequential dependencies and temporal trends. Despite these advances, many deep learning–based ESP studies focus primarily on binary failure prediction and rely on a single data modality, typically electrical or pressure telemetry, thereby underutilizing vibration and thermal information^23^.

### Transformer-based time-series models

More recently, transformer architectures have gained attention for time-series forecasting due to their self-attention mechanism, which enables efficient modeling of long-range dependencies without the limitations of recurrent structures^24^. Transformer-based models have demonstrated superior performance in various industrial forecasting tasks, including energy systems, manufacturing processes, and predictive maintenance applications^25^.

In the context of ESP systems, transformer-based approaches remain relatively underexplored. Existing studies that employ transformers typically treat ESP telemetry as a generic time-series forecasting problem, without incorporating domain-specific knowledge or multi-modal data fusion^26^. Furthermore, most transformer-based models focus on short-term forecasting or anomaly detection rather than explicit RUL estimation, which is essential for actionable maintenance decision-making.

### Physics-informed and hybrid prognostic models

A growing body of research has highlighted the limitations of purely data-driven models when applied to safety-critical and physically governed systems, where predictions must remain consistent with known physical behavior^27^. While deep learning approaches are effective in capturing complex nonlinear patterns, they often lack interpretability and may produce physically implausible outputs, particularly when extrapolating beyond the training data or under limited data availability. To address these challenges, physics-informed machine learning has emerged as a promising paradigm that integrates domain knowledge into data-driven models to enhance robustness, generalization, and physical consistency^27,28^.

In general, physics-informed approaches incorporate prior knowledge through several complementary mechanisms. First, governing equations derived from first principles such as conservation laws, thermodynamic relationships, or mechanical dynamics can be embedded directly into the model formulation, as demonstrated in physics-informed neural networks (PINNs)^27^. Second, soft constraints can be introduced by augmenting the loss function with penalty terms that discourage violations of known physical relationships, allowing flexible integration of physical knowledge without restricting model capacity^28^. Third, hybrid architectures can combine data-driven components with simplified physics-based models, enabling complementary learning of system dynamics and improving predictive reliability^28^. These strategies allow the model to learn complex patterns from data while ensuring adherence to underlying physical laws.

Physics-informed models have been successfully applied across a wide range of engineering domains, including fluid dynamics, turbomachinery, structural health monitoring, and energy systems, where they have demonstrated improved extrapolation capability, reduced sensitivity to noise, and enhanced interpretability^28^. In prognostics and health management applications, incorporating physical constraints has been shown to improve the reliability of RUL estimation by enforcing physically meaningful degradation trajectories and reducing unrealistic predictions under varying operating conditions^27,28^.

In the context of ESPs, several well-established physical principles govern system operation and degradation behavior. These include hydraulic affinity laws, which describe the relationships between flow rate, head, and power consumption; thermal balance equations, which characterize heat generation and dissipation within the motor; and vibration-based degradation mechanisms associated with faults such as bearing wear, imbalance, and shaft misalignment^29^. These relationships provide valuable prior knowledge that can guide predictive models toward physically consistent behavior, particularly under dynamic and harsh operating conditions.

Despite these advances, the integration of physics-informed learning into ESP predictive maintenance remains limited^30^. Most existing ESP prognostic models rely primarily on purely data-driven approaches and do not explicitly enforce physical consistency constraints, such as adherence to pump operating curves, thermodynamic limits, or monotonic degradation trends in vibration signals^29,31^. As a result, model predictions may violate known operational principles, reducing trust, interpretability, and reliability in real-world deployment scenarios^31^. This limitation highlights the need for hybrid modeling frameworks that combine the strengths of data-driven learning with domain-specific physical knowledge to achieve robust and interpretable ESP prognostics.

Recent advances in RUL prediction have increasingly focused on integrating deep learning with uncertainty-aware modeling, physics-informed learning, and operational decision optimization. For example, recent studies have proposed deep learning–stochastic ensemble frameworks that combine transformer-based architectures, stochastic degradation modeling, and dynamic mission abort policies to improve predictive maintenance decision-making under uncertain operational conditions^32^. These approaches demonstrate that integrating uncertainty estimation with prognostic modeling can significantly enhance the reliability of maintenance planning in safety-critical systems.

In parallel, physics-informed Bayesian frameworks and digital twin approaches have emerged as promising strategies for safety-constrained prognostics and control. Recent studies on battery systems have shown that Bayesian PINNs and uncertainty-aware digital twins can improve RUL prediction reliability by integrating physical degradation mechanisms with probabilistic inference and safety-aware control policies^33^. Such approaches are particularly relevant for industrial prognostics where uncertainty quantification and operational safety are critical.

Furthermore, recent research has explored the integration of deep learning ensembles with operational optimization strategies, including dynamic burn-in optimization for lithium-ion batteries. These methods combine ensemble-based RUL prediction with adaptive operational decision-making to optimize reliability, maintenance cost, and system safety simultaneously^34^. Collectively, these studies highlight a broader transition from purely predictive models toward integrated prognostic frameworks that jointly address degradation modeling, uncertainty quantification, and operational decision support.

Despite these advances, the application of such uncertainty-aware and physics-informed prognostic frameworks to ESP systems remains limited. In particular, few studies have explored the integration of domain-specific physical constraints, multi-modal transformer-based learning, and joint failure prediction and RUL estimation within a unified framework for ESP predictive maintenance.

To systematically compare existing ESP predictive maintenance approaches, **Table **2 summarizes representative studies in terms of data sources, modeling strategies, data modalities, and prognostic capabilities.

**Table 2:** Comparative Review of Existing ESP Predictive Maintenance Models.

Study/Method	Data Modalities	Model Type	Multi-Modal	Physics-Informed	Physics Constraint Type	Failure Prediction	RUL Estimation	Multi-Task Learning	Strengths	Limitations
Abdelaziz et al. (2017)^35^	Telemetry	Random Forest	No	No	–	Yes	No	No	Simple, interpretable	Weak nonlinear modeling
Jansen et al. (2019)^31^	Basic metrics	SVM/DT	No	No	–	Yes	No	No	Fast	Low accuracy
AlBallam (2022)^30^	Operational data	LSTM	No	No	–	Yes	No	No	Temporal modeling	Single modality
Abdalla et al. (2023)^1^	Electrical + pressure	CNN	Partial	No	–	Yes	No	No	Good fault detection	No degradation modeling
TimeGPT (2023)^10^	Time-series	Transformer	No	No	–	Yes	No	No	Long-range modeling	No domain knowledge
Time-LLM (2023)^8^	Time-series	LLM + Transformer	No	No	–	Yes	No	No	Handles complex sequences	Heavy computation
Physics-informed models (other domains)	Multi-sensor	PINN/Hybrid DL	Yes	Yes	Governing equations + loss constraints	Yes	Yes	Partial	Physically consistent	Not ESP-specific
HF-ESPNet (This Work)	Telemetry + Vibration + Thermal + Metadata + Logs	Hybrid Transformer + Autoencoder	Yes	Yes	Affinity laws + thermal balance + vibration constraints (loss-based)	Yes	Yes	Yes	Multi-modal + physics-informed + RUL + interpretable	Higher complexity

As shown in **Table **2, most existing approaches rely on single-modality telemetry data and focus primarily on failure classification, while multi-modal fusion, physics-informed modeling, and explicit RUL estimation remain largely unexplored. These limitations motivate the development of the proposed framework.

While recent advances in transformer-based and physics-informed machine learning have demonstrated strong performance in time-series forecasting and prognostics across various engineering domains, most existing approaches are either purely data-driven or incorporate generic physics constraints without domain-specific adaptation. In particular, prior transformer-based models such as TimeGPT and Time-LLM focus on sequence modeling but do not integrate multi-modal industrial data or enforce physical consistency. Similarly, physics-informed models developed in other domains often rely on governing equations specific to fluid dynamics or structural systems, and are not directly applicable to ESP operations without significant adaptation.

In contrast, the proposed HF-ESPNet framework introduces several key distinctions. First, it integrates heterogeneous ESP data sources, including electrical telemetry, vibration-derived features, thermal indicators, and well-level metadata, within a unified multi-modal transformer architecture. Second, it incorporates domain-specific physics-informed constraints tailored to ESP systems, including hydraulic affinity relationships, thermal behavior, and vibration-based degradation patterns, rather than relying on generic physical regularization. Third, the model adopts a multi-task learning strategy that jointly performs failure prediction and RUL estimation, enabling simultaneous classification and regression within a single framework. These combined characteristics distinguish the proposed approach from existing transformer-based and physics-informed models in both methodology and application scope.

### Identified research gaps

Based on the reviewed literature, several key gaps can be identified. First, there is a lack of unified frameworks that integrate multi-modal ESP data including telemetry, vibration, thermal indicators, and operational metadata within a single predictive model. Second, most existing approaches emphasize failure classification rather than continuous degradation modeling and RUL estimation. Third, the majority of data-driven models remain purely statistical and do not incorporate physics-informed constraints, limiting interpretability and generalization. Finally, transformer-based architectures, despite their potential, have not been fully explored in ESP prognostics, particularly in combination with domain knowledge and multi-modal data fusion.

###  Contribution of this study

Although recent advances in transformer-based time-series modeling and physics-informed machine learning have demonstrated strong performance across various engineering domains, their integration within the context of ESP predictive maintenance remains limited and insufficiently adapted to domain-specific requirements. In particular, existing approaches either rely on purely data-driven architectures without enforcing physical consistency, or incorporate generic physics-based regularization that does not adequately reflect the operational characteristics of ESP systems.

To address these limitations, this study proposes a unified framework that integrates multi-modal data fusion, transformer-based temporal modeling, and domain-specific physics-informed learning for ESP prognostics. The novelty of the proposed HF-ESPNet framework lies in the following key contributions:

1. Domain-specific physics-informed modeling for ESP systems:

Unlike existing physics-informed approaches that rely on generic formulations, this work incorporates ESP-specific physical principles including hydraulic affinity relationships, thermal behavior, and vibration-based degradation mechanisms into the learning process. These constraints are embedded directly into the optimization objective, enforcing physically consistent predictions and improving model reliability under varying operating conditions.

2. Multi-modal representation learning through structured feature fusion:

The proposed model integrates heterogeneous data sources, including electrical telemetry, vibration-derived features, thermal indicators, and well-level metadata, using modality-specific encoders followed by a structured fusion mechanism. This design preserves modality-dependent characteristics while enabling effective cross-modal interaction, allowing the model to capture complex degradation patterns that are not observable from single-modality data.

3. Joint failure prediction and Remaining Useful Life estimation:

The framework adopts a multi-task learning formulation that simultaneously performs failure classification and RUL regression. This joint modeling strategy enables the learning of shared degradation representations across discrete and continuous prognostic tasks, providing both early warning capabilities and continuous health assessment within a single unified model.

4. Structured integration of physics-informed constraints within the learning objective:

The proposed framework introduces a physics-informed loss formulation in which domain-specific constraints are incorporated as structured regularization terms. This approach effectively constrains the hypothesis space of the model to physically consistent solutions, distinguishing it from purely data-driven models and loosely regularized physics-informed approaches.

The proposed HF-ESPNet framework differs from several existing transformer-based approaches in both architectural design and learning formulation. Compared to the Temporal Fusion Transformer (TFT), which is primarily designed for structured tabular time-series forecasting, the proposed model explicitly supports heterogeneous multi-modal inputs, including vibration-derived features and operational metadata, which are not directly handled within standard TFT architectures.

In contrast to conventional multi-modal transformer models that typically rely on simple feature concatenation or shared embedding representations, the proposed framework employs modality-specific encoders followed by structured feature fusion, enabling improved representation learning and preservation of modality-specific characteristics.

Furthermore, while recent studies have explored the integration of physics-informed regularization within deep learning models, such approaches often rely on generic physical constraints or domain-independent formulations. In this work, physics-informed constraints are derived specifically from ESP operating principles and are systematically incorporated into the training objective, resulting in physically consistent and domain-adaptive predictions.

Finally, unlike most existing transformer-based prognostic models that focus on a single predictive task, the proposed framework adopts a multi-task learning formulation that jointly models failure prediction and RUL estimation. This joint approach enhances the model’s ability to capture both discrete failure events and continuous degradation dynamics.

From a methodological perspective, the proposed approach can be interpreted as a constrained multi-task learning framework in which the hypothesis space is regularized by domain-specific physical laws while jointly optimizing discrete failure classification and continuous degradation modeling.

These contributions collectively distinguish the proposed HF-ESPNet framework from existing data-driven, transformer-based, and physics-informed models, both in terms of methodological innovation and application-specific design for ESP predictive maintenance.

## Methodology

This section describes the methodological framework adopted in this study, including the characteristics of the ESP operational dataset, data preprocessing procedures, model architecture, training strategy, and evaluation metrics. The proposed methodology is designed to ensure reproducibility, physical consistency, and robust performance assessment under realistic operating conditions.

### ESP operational dataset description

The dataset employed in this study consists of historical ESP operational measurements collected from upstream oil and gas production environments. It comprises more than 58,000 multivariate time-series samples representing the full operational lifecycle of ESP systems, including normal operation, progressive degradation, and failure events. The dataset captures realistic operating conditions observed over extended time periods, reflecting variations in load, environmental conditions, and system aging.

The data include multiple sensing modalities recorded by standard ESP monitoring systems, encompassing electrical, hydraulic, thermal, and mechanical indicators, as well as contextual well metadata and maintenance records^14^. Electrical telemetry includes motor current measurements, which reflect load conditions and electrical stress. Hydraulic behavior is characterized through intake and discharge pressure signals, providing insight into flow conditions and pump efficiency^2^. Thermal conditions are monitored using motor temperature measurements and derived thermal indicators, capturing heat generation and dissipation patterns. Mechanical health is represented through vibration-derived features obtained from frequency-domain analysis, which are particularly sensitive to faults such as bearing wear and misalignment.

In addition to dynamic sensor data, the dataset includes static well-level metadata such as pump configuration, installation depth, fluid properties, and gas–oil ratio. These variables provide important contextual information that supports modeling of operational variability across different wells and production conditions. Maintenance and failure logs document intervention events, failure causes, and service dates, enabling the construction of supervised labels for failure prediction and the derivation of RUL targets.

To improve transparency, the dataset contains operational records from multiple ESP units deployed across different wells, including several tens of documented failure events spanning a range of degradation mechanisms. The monitoring duration for individual ESP units varies from several months to multiple years, depending on operational conditions and maintenance schedules. This diversity ensures that the dataset captures both short-term operational fluctuations and long-term degradation behavior.

The recorded failure events correspond to multiple degradation mechanisms, including mechanical faults (e.g., bearing wear and shaft misalignment), hydraulic instabilities (e.g., gas lock and flow disruption), and electrical degradation phenomena (e.g., insulation breakdown and thermal overload), as discussed in Sect. 2.1. These failure events are used to define degradation trajectories and generate RUL labels based on the time remaining until the next recorded failure. Following each maintenance intervention, the system is assumed to return to a healthy operational state, and the RUL is reset accordingly. As a result, the dataset contains multiple degradation cycles, enabling the model to learn temporal patterns across repeated failure–repair sequences.

It is important to note that the failure labels correspond to a finite set of observed failure modes documented in maintenance records. Consequently, the supervised components of the proposed framework are primarily trained to recognize degradation patterns associated with these known failure types. However, the model also learns continuous degradation representations through multi-modal temporal modeling and RUL estimation, enabling it to capture general degradation trends beyond specific labeled failure modes. In particular, the integration of heterogeneous data sources allows the model to identify anomalous behavior and progressive deterioration, even in the absence of explicit failure labels. Nevertheless, the ability to accurately classify completely unseen failure modes may be limited, and further validation using broader datasets is required to assess generalization to novel degradation scenarios.

Representative signal behavior prior to failure includes gradual increases in motor current and temperature, increased variability in pressure signals, and growth in vibration energy within characteristic frequency bands. These trends are consistent with known ESP degradation mechanisms and confirm that the dataset captures realistic multi-modal degradation dynamics suitable for predictive maintenance and RUL estimation.

To ensure data confidentiality and compliance with industrial data-sharing constraints, all records were anonymized while preserving the statistical properties, temporal dependencies, and degradation characteristics necessary for predictive maintenance analysis. While raw data cannot be publicly released due to proprietary restrictions, the dataset structure, feature composition, and degradation characteristics are described in sufficient detail to support reproducibility and methodological transparency.

A structured summary of the dataset composition, representative variables, and the role of each data modality in the prognostic framework is provided in **Table **3.

**Table 3:** Summary of ESP Operational Dataset and Its Role in Prognostic Modeling.

Data Modality	Representative Variables	Sampling/Resolution	Unit	Role in Prognostics	Relation to Failure Modeling
Time-Series Telemetry	Motor current, intake pressure, discharge pressure, motor temperature	Uniformly resampled time series	A, PSI, °C	Captures electrical load, hydraulic behavior, and thermal stress	Reflects system response under degradation and pre-failure conditions
Vibration Indicators (FFT)	Spectral energy in 0–500 Hz frequency bands	Frequency-domain features derived from time windows	Hz (band energy)	Identifies mechanical degradation such as bearing wear, imbalance, and misalignment	Sensitive to early-stage mechanical faults and progressive wear patterns
Thermal Degradation Features	Heating rate, cooling efficiency, temperature gradients	Derived temporal features	°C/min	Models long-term thermal ageing and insulation degradation	Indicates cumulative degradation and approaching failure states
Well Metadata	Pump type, installation depth, fluid properties, gas–oil ratio	Static/categorical	–	Supports model generalization across wells	Provides contextual variability but not direct failure labeling
Maintenance & Failure Logs	Failure cause, repair type, last service date	Event-based records	–	Provides ground truth labels for failure prediction and RUL construction	Defines observed failure modes and RUL targets; limited to recorded failure types

As shown in **Table **3, while maintenance logs provide discrete failure labels, the remaining data modalities contribute to continuous degradation modeling, enabling the framework to learn temporal health evolution beyond explicitly labeled failure events.

### Data preprocessing and feature construction

Prior to model training, a series of preprocessing and feature construction steps were applied to ensure data quality, consistency, and compatibility across ESP units operating under diverse conditions. Raw telemetry signals were first synchronized and resampled to a uniform temporal resolution to enable consistent temporal modeling. Missing values were handled using linear interpolation for short-duration gaps and forward-filling for stable operational intervals. Outliers resulting from sensor faults or communication errors were identified using statistical thresholding and removed to prevent bias in model training.

To account for differences in measurement units and operating ranges among electrical, hydraulic, and thermal variables, all continuous time-series features were normalized using min–max scaling, defined as:

1 $$\:{x}^{{\prime\:}}=\frac{x-{x}_{\mathrm{m}\mathrm{i}\mathrm{n}}}{{x}_{\mathrm{m}\mathrm{a}\mathrm{x}}-{x}_{\mathrm{m}\mathrm{i}\mathrm{n}}}$$

where * x*denotes the original feature value, and $\:{x}_{\mathrm{m}\mathrm{i}\mathrm{n}}$and $\:{x}_{\mathrm{m}\mathrm{a}\mathrm{x}}$represent the minimum and maximum values observed in the training data, respectively. This normalization improves numerical stability and accelerates convergence during model optimization^36^.

Vibration signals were transformed from the time domain into the frequency domain using the Fast Fourier Transform (FFT), expressed as^37^:

2 $$\:X\left(f\right)=\sum\:_{n=0}^{N-1}x\left(n\right){\hspace{0.17em}}{e}^{-j2\pi\:fn/N}$$

where $\:x\left(n\right)$denotes the vibration signal at time index * n*, * N*is the total number of samples, and $\:X\left(f\right)$represents the complex frequency spectrum. Energy-based features were subsequently extracted over predefined frequency bands associated with mechanical degradation mechanisms, such as bearing wear and imbalance.

Thermal degradation indicators were constructed by computing temperature gradients and heating rates over time, enabling the model to capture long-term ageing effects and insulation degradation. Categorical well metadata, including pump configuration and operating context, were encoded using embedding representations to facilitate their integration with numerical features within the unified learning framework.

#### **Remaining useful life definition and data partitioning strategy**

To support RUL estimation, failure events recorded in maintenance logs were used as reference points for constructing degradation targets^38^. For each time step * t*, the RUL is defined as the remaining operational time until the next recorded failure event associated with the same ESP unit:

3 $$\:\mathrm{RUL}\left(t\right)={t}_{\mathrm{failure}}-t$$

where $\:{t}_{\mathrm{failure}}$ denotes the timestamp of the subsequent failure event. In this study, RUL is expressed in discrete time steps corresponding to the uniformly resampled time-series data. Each time step represents a fixed temporal interval derived from the original telemetry acquisition frequency, enabling consistent degradation modeling across different ESP units and operational conditions.

This formulation enables the construction of continuous degradation trajectories leading up to failure events. The failure threshold is defined by the occurrence of a recorded failure or maintenance intervention event, at which point the ESP system is considered to have reached the end of its useful operational life. Following maintenance or repair, the system is assumed to return to a healthy operational state, and the RUL is reset, initiating a new degradation cycle. Consequently, the dataset captures multiple degradation cycles across repeated failure–repair sequences, supporting robust temporal learning of progressive degradation behavior.

To prevent bias toward excessively long degradation trajectories and improve regression stability, RUL values were capped at a maximum threshold of RUL_max = 200-time steps during training. This prevents extreme target values from dominating the optimization process and improves numerical stability in long operational sequences.

Sequences that do not terminate in a recorded failure event are treated as right-censored data. These censored sequences are excluded from supervised RUL regression targets but still contribute to representation learning within the multi-modal temporal modeling framework. Partial degradation cycles, including sequences that capture only early-stage or intermediate degradation behavior, are retained to improve the model’s generalization across different degradation stages and operational scenarios.

Unlike approaches relying on predefined health indices or manually engineered degradation curves, the proposed framework implicitly learns degradation representations directly from heterogeneous multi-modal time-series data through transformer-based temporal modeling. This allows the model to capture complex and non-linear degradation behavior while maintaining direct correspondence between RUL targets and operational failure events recorded in maintenance logs.

**Fig. 1:**
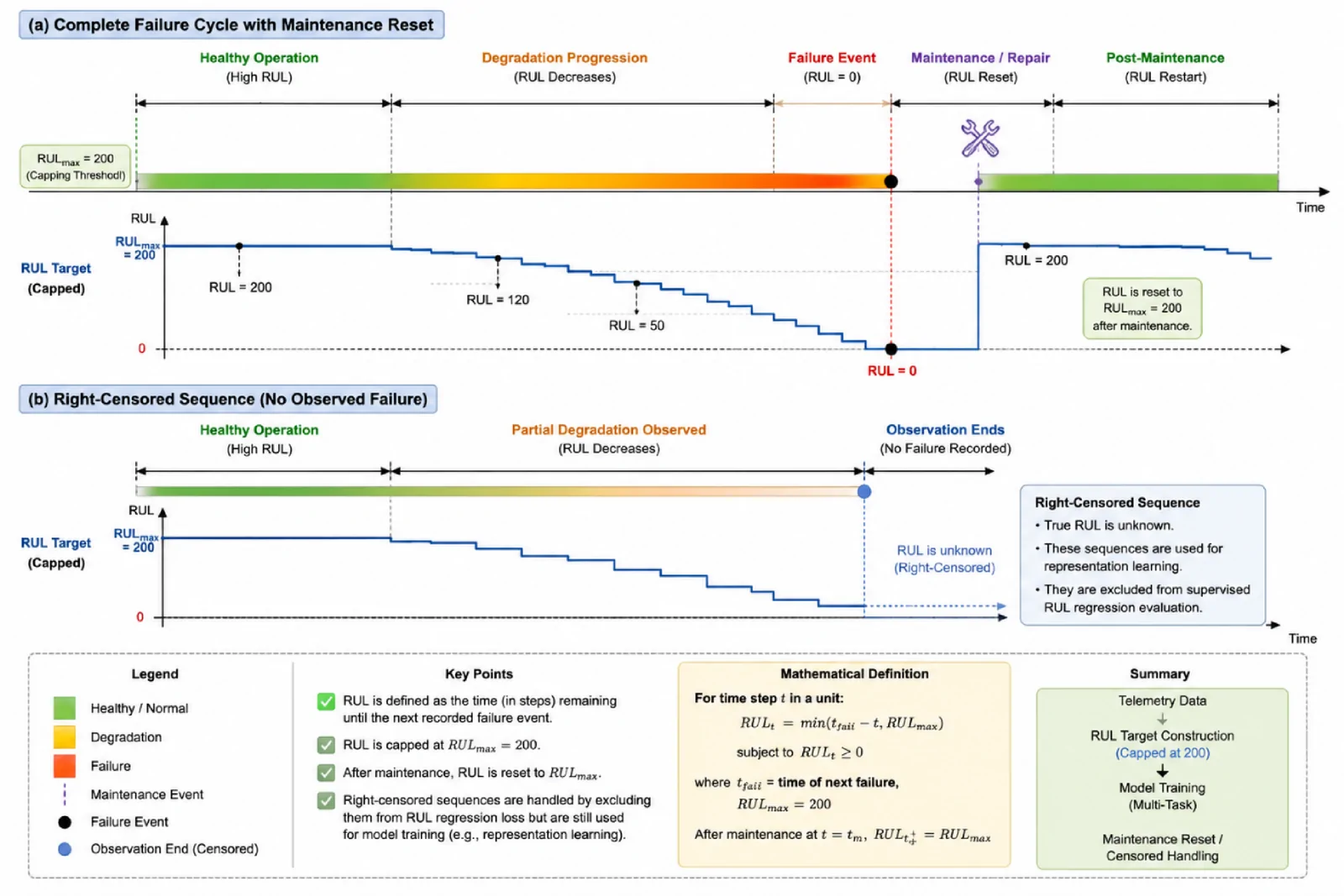
Schematic illustration of RUL target construction including degradation progression, failure-event definition, maintenance reset mechanism, RUL capping, and handling of right-censored operational sequences within the proposed prognostic framework.

A schematic illustration of the RUL target construction process, including maintenance reset, RUL capping, and handling of right-censored operational sequences, is provided in **Fig. **1 for clarity.

### Training, validation, and testing strategy

To ensure unbiased performance evaluation and prevent information leakage, the dataset was partitioned into training, validation, and testing subsets using a time-aware splitting strategy. Operational data from earlier time periods were assigned to the training set, while data from subsequent periods were reserved for validation and testing^39^. This temporal partitioning reflects realistic deployment conditions in which models are trained on historical data and evaluated on future, unseen operational behavior.

Approximately 70% of the dataset was used for training, 15% for validation, and the remaining 15% for testing. The training set was utilized to optimize model parameters, while the validation set supported hyperparameter tuning and early stopping. The test set, which was not accessed during training or validation, was used exclusively for final performance evaluation, ensuring an unbiased assessment of model generalization.

A summary of the dataset partitioning strategy is provided in Table 4, which outlines the proportion of data assigned to each subset and their respective roles in the model development process.

**Table 4:** Summary of Dataset Partitioning for Model Development and Evaluation.

Subset	Percentage of Data	Operational Time Period	Purpose
Training Set	70%	Early operational stages	Model parameter learning and representation training
Validation Set	15%	Intermediate operational stages	Hyperparameter tuning and early stopping
Test Set	15%	Late operational stages (unseen data)	Final performance evaluation and generalization assessment

This partitioning strategy ensures that the reported results accurately reflect the model’s ability to generalize across diverse operating conditions and varying stages of ESP degradation.

### Model architecture

The proposed HF-ESPNet model is a hybrid multi-modal architecture that integrates modality-specific encoders, a sensor fusion layer, a transformer-based temporal modeling block, and physics-informed constraints. Each data modality is processed by a dedicated encoder designed to capture its unique characteristics. The encoded representations are fused into a unified latent space, which is then passed through a transformer encoder to model long-range temporal dependencies^40^.

To enhance physical consistency, physics-informed constraints derived from ESP operating principles such as pump affinity relationships, thermal behavior, and vibration growth patterns are incorporated into the learning process. The model jointly performs two predictive tasks: binary failure classification and Remaining Useful Life regression, enabling both early warning and time-to-failure estimation^41^.

From a methodological perspective, the novelty of the proposed HF-ESPNet framework lies in the integration of three complementary components within a unified learning architecture. First, unlike standard transformer-based models that operate on single-modality time-series data, the proposed approach employs modality-specific encoders to process heterogeneous inputs, followed by a structured fusion mechanism that preserves cross-modal interactions. Second, the model incorporates domain-specific physics-informed constraints derived from ESP operating principles, which are embedded into the training objective as structured regularization terms rather than generic physical priors. Third, the framework adopts a multi-task learning formulation that jointly optimizes failure classification and RUL regression, enabling the model to learn shared degradation representations across discrete and continuous prognostic tasks.

This combination of multi-modal data fusion, domain-specific physics integration, and joint prognostic modeling distinguishes the proposed approach from existing transformer-based, physics-informed, and prognostic models, both architecturally and mathematically.

**Fig. 2:**
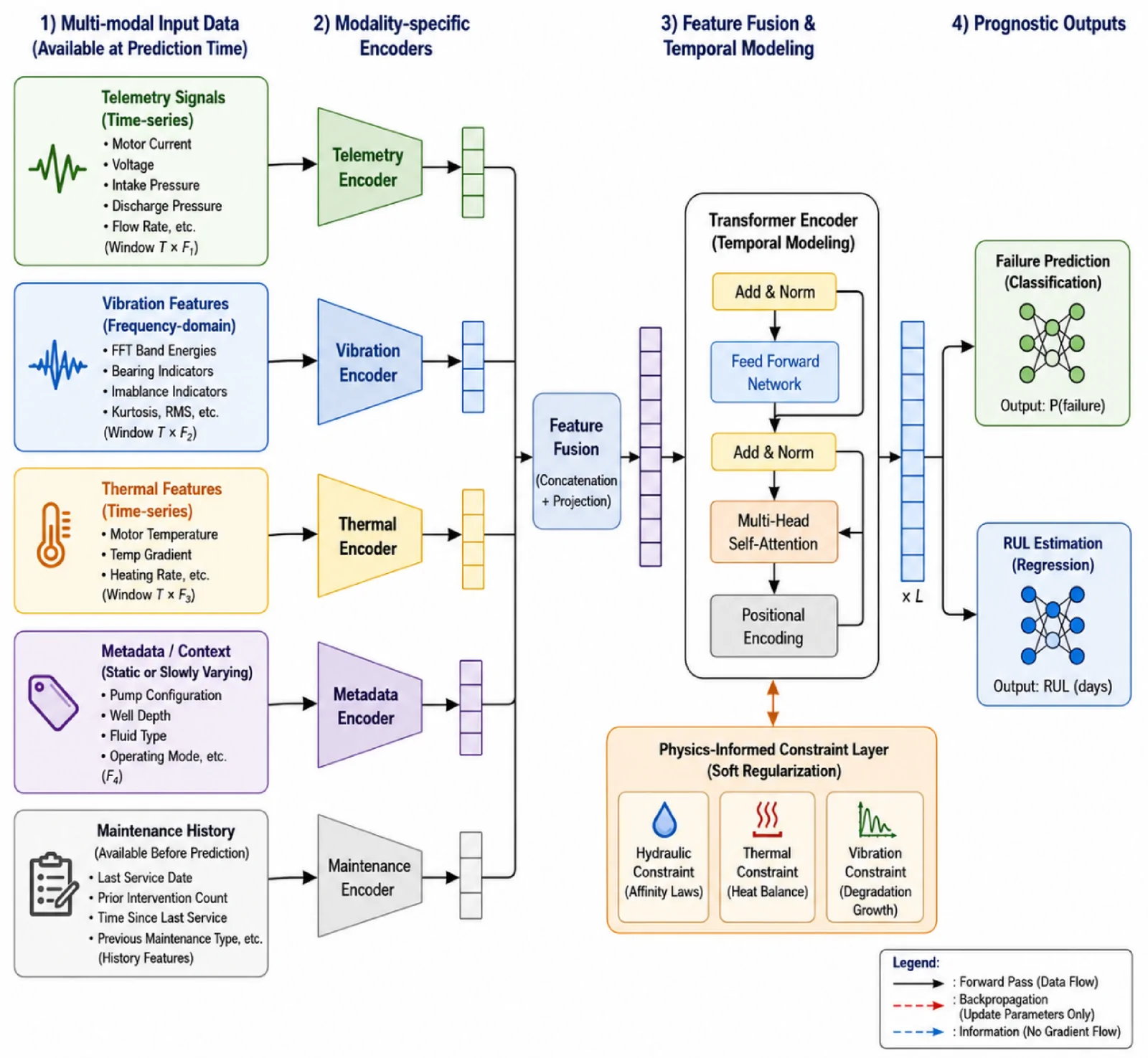
Detailed architecture of the proposed HF-ESPNet framework.

To improve clarity and reproducibility, a detailed architectural representation of the proposed HF-ESPNet model is provided in **Fig. **2. Unlike the conceptual workflow diagrams presented earlier, this figure illustrates the internal structure of the model, including modality-specific encoding layers, feature fusion mechanism, transformer-based temporal modeling, and the integration of physics-informed constraints within the learning framework. The model processes heterogeneous input data through dedicated encoders, which are then combined into a unified latent representation and passed through transformer layers to capture temporal dependencies. Finally, the shared representation is used for dual-task prediction, including failure classification and Remaining Useful Life estimation.

Figure 2. Detailed architecture of the proposed HF-ESPNet framework, illustrating modality-specific encoders for multi-modal inputs (telemetry, vibration, thermal features, and metadata), feature fusion layer, transformer-based temporal modeling, physics-informed constraint integration, and dual output heads for failure prediction and RUL estimation.

#### Theoretical Background of the Transformer-Based Prognostic Model

Transformer architectures are designed to model sequential data by capturing long-range dependencies through self-attention mechanisms rather than recurrent connections. Unlike recurrent neural networks, which process sequences sequentially and may suffer from vanishing gradients and limited memory, transformers operate on entire sequences simultaneously, enabling efficient learning of temporal relationships across long operating histories^5,42^.

Given an input sequence of embedded feature vectors, the transformer computes query (Q), key (K), and value (V) representations through learned linear projections. The self-attention mechanism evaluates the relevance of each time step with respect to all others by computing a weighted sum of value vectors, where the weights are determined by the similarity between queries and keys. This process allows the model to dynamically focus on critical degradation periods, such as early anomaly onset or pre-failure transitions.

Mathematically, scaled dot-product attention is defined as^21^:

4 $$\:\mathrm{Attention}(Q,K,V)=\mathrm{softmax}\left(\frac{Q{K}^{t{\top\:}}}{\sqrt{{d}_{k}}}\right)V$$

where $\:{d}_{k}$denotes the dimensionality of the key vectors. Multi-head attention extends this mechanism by applying multiple attention operations in parallel, enabling the model to capture diverse temporal patterns associated with different degradation mechanisms.

In the context of ESP predictive maintenance, this capability is particularly valuable because degradation processes are non-linear, multi-stage, and influenced by interacting electrical, mechanical, and thermal factors. The transformer encoder in HF-ESPNet enables the model to learn both short-term fluctuations and long-term degradation trends, improving failure prediction reliability and Remaining Useful Life estimation accuracy.

To enhance physical plausibility and robustness, the transformer output is further constrained through physics-informed regularization, ensuring that learned representations remain consistent with known ESP operating principles. This hybrid formulation combines data-driven temporal modeling with domain knowledge, addressing key limitations of purely statistical approaches^41^.

**Fig. 3:**
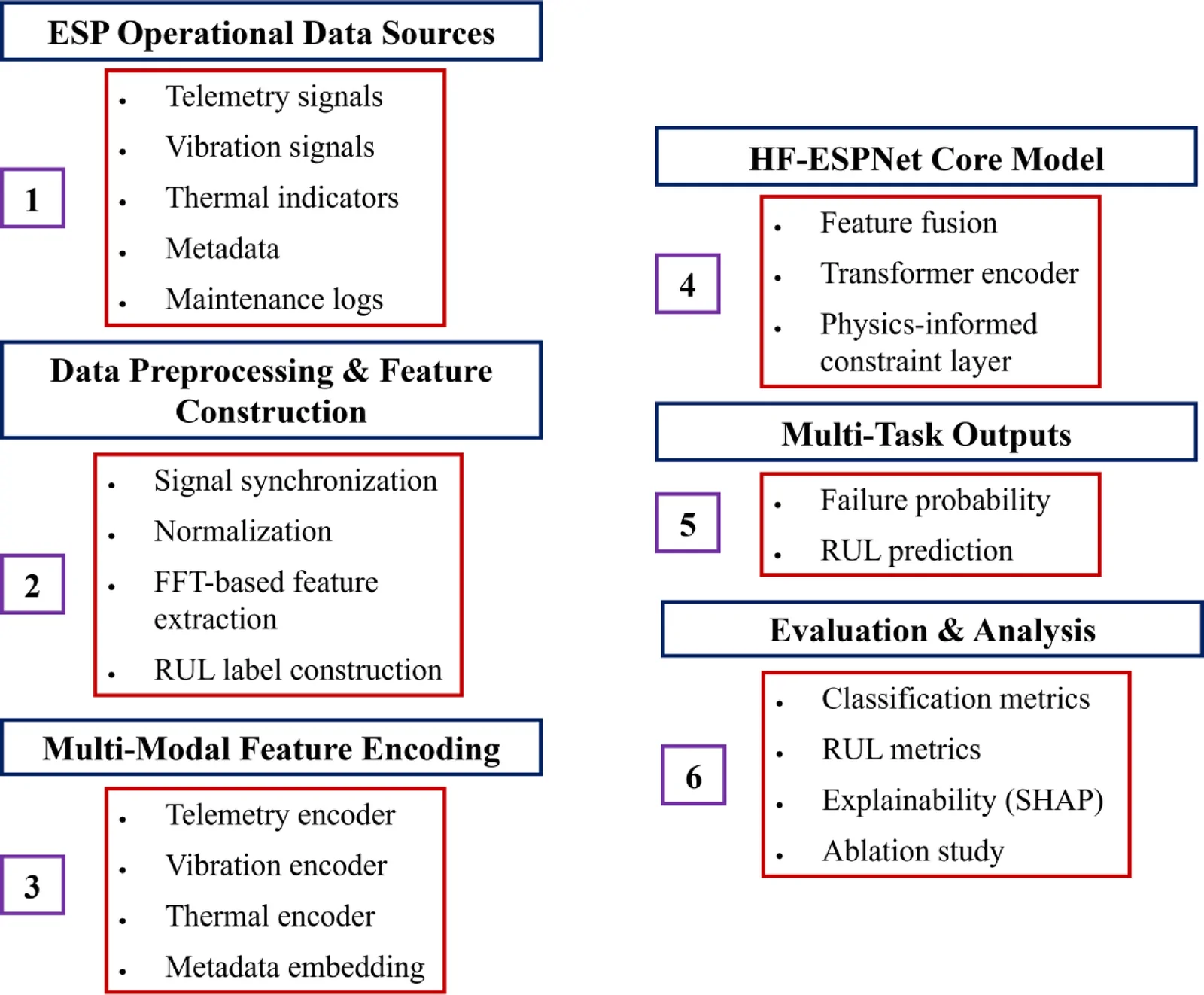
Conceptual workflow and architecture of the proposed HF-ESPNet-based predictive maintenance framework.

Figure 3. End-to-end workflow of the proposed study, illustrating ESP data acquisition, preprocessing, multi-modal feature encoding, transformer-based physics-informed modeling, and performance evaluation for failure prediction and Remaining Useful Life estimation.

### Model training and optimization

The proposed HF-ESPNet framework is trained using a multi-task learning strategy that jointly optimizes ESP failure prediction and RUL estimation. This joint formulation enables the model to learn shared latent representations that capture both discrete failure events and continuous degradation trajectories, thereby improving prognostic accuracy, robustness, and generalization across varying operating conditions.

Failure prediction is formulated as a binary classification task and optimized using binary cross-entropy loss, while RUL estimation is treated as a regression problem and optimized using mean squared error (MSE). In addition to these data-driven objectives, domain-specific physics-informed constraints are incorporated into the learning process to enforce physically consistent predictions based on known ESP operating principles.

#### Physics-informed constraint formulation

To ensure physically consistent prognostic predictions, the proposed HF-ESPNet framework incorporates domain-specific physical constraints derived from ESP operating principles. These constraints are implemented as soft penalty terms within the optimization objective, allowing the model to maintain flexibility while discouraging physically implausible predictions.

(i) Hydraulic Affinity Constraint.

The hydraulic behavior of ESP systems is governed by classical pump affinity laws, which describe the relationship between pump rotational speed * N*, flow rate * Q*, head * H*, and power * P*:

5 $$\:\frac{{Q}_{1}}{{Q}_{2}}=\frac{{N}_{1}}{{N}_{2}},\frac{{H}_{1}}{{H}_{2}}={\left(\frac{{N}_{1}}{{N}_{2}}\right)}^{2},\frac{{P}_{1}}{{P}_{2}}={\left(\frac{{N}_{1}}{{N}_{2}}\right)}^{3}$$.

In this study, hydraulic consistency is enforced using operational variables including motor current, intake pressure, discharge pressure, and estimated flow-related behavior derived from telemetry data. The hydraulic loss term is formulated as:

6 $$\:{\mathcal{L}}_{hyd}=\frac{1}{M}{\sum\:}_{i=1}^{M}{\mid\:{\widehat{H}}_{i}-{H}_{i}^{phys}\mid\:}^{2}$$.

where $\:{\widehat{H}}_{i}$represents the predicted hydraulic state and $\:{H}_{i}^{phys}$denotes the physically expected behavior derived from affinity relationships.

(ii) Thermal Balance Constraint.

The thermal dynamics of the ESP motor are modeled using a simplified heat balance relationship:

7 $$\:\frac{dT}{dt}=\frac{{P}_{loss}-{Q}_{cool}}{{C}_{th}}$$.

where:

- * T*denotes motor temperature,
- $\:{P}_{loss}$represents electrical and mechanical losses,
- $\:{Q}_{cool}$denotes cooling effects associated with fluid circulation,
- $\:{C}_{th}$is the effective thermal capacity.

The thermal constraint penalizes physically inconsistent temperature evolution trajectories:

8 $$\:{\mathcal{L}}_{thermal}=\frac{1}{M}{\sum\:}_{i=1}^{M}{\mid\:\frac{d{\widehat{T}}_{i}}{dt}-\frac{{P}_{loss,i}-{Q}_{cool,i}}{{C}_{th}}\mid\:}^{2}$$.

Motor temperature, current measurements, and thermal degradation indicators are used to estimate these dynamics.

(iii) Vibration-Based Degradation Constraint.

Mechanical degradation in ESP systems is reflected through progressive growth of vibration energy within characteristic frequency bands associated with bearing wear, imbalance, and shaft misalignment. Vibration degradation is modeled using frequency-domain features extracted through Fast Fourier Transform (FFT):

9 $$\:{E}_{band}={\sum\:}_{f={f}_{1}}^{{f}_{2}}\mid\:X\left(f\right){\mid\:}^{2}$$.

To enforce physically meaningful degradation progression, monotonic growth behavior is encouraged during degradation trajectories:

10 $$\:{\mathcal{L}}_{vib}=\frac{1}{M}{\sum\:}_{i=1}^{M}\mathrm{m}\mathrm{a}\mathrm{x}\left(0,-\frac{d{E}_{band,i}}{dt}\right)$$.

This formulation penalizes unrealistic decreases in degradation-sensitive vibration energy during failure progression.

#### Physics-informed loss integration

The overall training objective combines classification, regression, and physics-informed consistency terms:

11 $$\:{L}_{\mathrm{total}}={\lambda\:}_{1}{L}_{\mathrm{cls}}+{\lambda\:}_{2}{L}_{\mathrm{rul}}+{\lambda\:}_{3}{L}_{\mathrm{hyd}}+{\lambda\:}_{4}{L}_{\mathrm{thermal}}+{\lambda\:}_{5}{L}_{\mathrm{vib}}$$

where:

- $\:{L}_{\mathrm{cls}}$: binary cross-entropy loss for failure classification.
- $\:{L}_{\mathrm{rul}}$: mean squared error loss for RUL estimation.
- $\:{L}_{\mathrm{hyd}}$, $\:{L}_{\mathrm{thermal}}$, and $\:{L}_{\mathrm{vib}}$: hydraulic, thermal, and vibration-based physics-informed constraints, respectively.

The weighting coefficients $\:{\lambda\:}_{1}$-$\:{\lambda\:}_{5}$ were treated as tunable hyperparameters and selected empirically through validation-based optimization to balance predictive accuracy, Remaining Useful Life estimation, and physical consistency. The final values used during training were:

- $\:{\lambda\:}_{1}=1.0$ for classification loss.
- $\:{\lambda\:}_{2}=0.5$ for RUL regression loss.
- $\:{\lambda\:}_{3}=0.2$ for hydraulic consistency loss.
- $\:{\lambda\:}_{4}=0.2$ for thermal consistency loss.
- $\:{\lambda\:}_{5}=0.3$ for vibration consistency loss.

These weighting coefficients were selected after evaluating multiple combinations on the validation dataset to achieve stable convergence and balanced optimization performance across both prognostic tasks.

Analysis of the optimization process indicated that the classification loss contributed most strongly to early failure detection performance, whereas the RUL regression loss primarily improved temporal degradation forecasting accuracy. The physics-informed regularization terms mainly contributed to smoother RUL trajectories, improved physical consistency of degradation behavior, and enhanced model generalization under varying operational conditions and noisy industrial telemetry data.

All physical constraints are implemented as soft regularization terms rather than hard architectural constraints, allowing the framework to remain flexible under partially observed and uncertain operating conditions. Physical constants and scaling coefficients are normalized using operational ranges derived from the training dataset. Where direct physical measurements are unavailable, effective parameters are estimated from telemetry-derived quantities and treated as fixed during training rather than as learnable model parameters.

#### Optimization strategy

Model optimization is performed using the AdamW optimizer, which combines adaptive gradient updates with weight decay regularization to improve generalization and mitigate overfitting. A fixed learning rate of $\:1\times\:{10}^{-4}$ is used, and training is conducted using mini-batch gradient descent with a batch size of 64 samples.

The model is trained for a maximum of 60 epochs, with early stopping applied based on validation loss to prevent overfitting and ensure optimal parameter selection. This strategy enables efficient convergence while maintaining robustness to noise and variability in operational data.

To ensure a fair and unbiased comparison, all baseline models are trained using the same data partitions, optimization settings, and stopping criteria. This consistent experimental setup ensures that performance differences are attributable to model architecture and learning strategy rather than variations in training configuration.

#### Hyperparameter tuning and model configuration

To ensure reproducibility and fairness in model comparison, all baseline models and the proposed HF-ESPNet framework were optimized using validation-based hyperparameter tuning under identical data partitioning conditions. Hyperparameter selection was performed using grid-search and validation-loss monitoring, where the final configuration for each model was selected based on the best validation performance. The explored search ranges and final selected hyperparameters are summarized in **Table **5.

**Table 5:** Summary of Hyperparameter Search Ranges and Final Selected Configurations.

Model	Hyperparameter	Search Range	Final Value
Random Forest	Number of Trees	100–500	300
Random Forest	Maximum Depth	5–30	15
1D CNN	Learning Rate	1e − 4–1e − 2	1e − 3
1D CNN	Number of Layers	2–6	4
1D CNN	Hidden Dimension	32–256	128
LSTM	Learning Rate	1e − 4–1e − 2	1e − 3
LSTM	Hidden Dimension	64–256	128
LSTM	Number of Layers	1–4	2
TimeGPT	Learning Rate	1e − 5–1e − 3	5e − 4
TimeGPT	Attention Heads	2–8	4
TimeGPT	Transformer Layers	2–8	4
Time-LLM	Learning Rate	1e − 5–1e − 3	5e − 4
Time-LLM	Attention Heads	2–8	4
Time-LLM	Transformer Layers	2–8	4
HF-ESPNet	Learning Rate	1e − 5–1e − 3	1e − 4
HF-ESPNet	Hidden Dimension	64–256	128
HF-ESPNet	Transformer Layers	2–8	4
HF-ESPNet	Attention Heads	2–8	4
HF-ESPNet	Dropout Rate	0.1–0.5	0.2
HF-ESPNet	Optimizer	Adam/AdamW	AdamW

Early stopping with a patience of 10 epochs and batch size of 64 were consistently applied across all deep learning architectures. Sequence lengths between 20- and 100-time steps were evaluated during validation-based optimization. All models were trained using identical data partitions, preprocessing procedures, and evaluation criteria to ensure fair comparison.

### Evaluation metrics

Model performance was evaluated using established metrics appropriate for both classification and regression tasks. Failure prediction performance was assessed using accuracy, precision, recall, F1-score, and the area under the receiver operating characteristic curve (AUC-ROC). RUL estimation performance was evaluated using root mean squared error (RMSE) and mean absolute error (MAE). These metrics provide complementary insights into prediction accuracy, robustness, and practical reliability.

In addition, ablation studies were conducted to quantify the contribution of individual model components, and explainability analyses were performed to identify the most influential input features.

All performance evaluation metrics were expressed in **Table **6.

**Table 6:** Performance Evaluation Metrics and Definitions^43–45^.

Prediction Task	Metric	Formal Definition	Evaluation Objective
Failure Prediction	Accuracy	$\:\frac{TP+TN}{TP+TN+FP+FN}$	Measures the proportion of correctly classified operational states over all test samples
	Precision	$\:\frac{TP}{TP+FP}$	Evaluates the reliability of failure alarms by quantifying false positive rates
	Recall (Sensitivity)	$\:\frac{TP}{TP+FN}$	Assesses the model’s ability to correctly identify imminent ESP failures
	F1-score	$\:2\cdot\:\frac{\mathrm{Precision}\cdot\:\mathrm{Recall}}{\mathrm{Precision}+\mathrm{Recall}}$	Provides a balanced measure between missed failures and false alarms
	AUC–ROC	Area under the Receiver Operating Characteristic curve	Quantifies discrimination performance across varying decision thresholds
RUL Estimation	RMSE	$\:\sqrt{\frac{1}{N}\sum\:_{i=1}^{N}({\widehat{y}}_{i}-{y}_{i}{)}^{2}}$	Penalizes large deviations in RUL prediction, emphasizing late-stage accuracy
	MAE	$\:\mathrm{MAE}=\frac{1}{N}\sum\:_{i=1}^{N}\mid\:{\widehat{y}}_{i}-{y}_{i}\mid\:$	Measures the average absolute deviation between predicted and true RUL, providing an interpretable indicator of overall RUL prediction accuracy.

## Results and Discussion

This section presents the experimental results obtained using the proposed HF-ESPNet framework and provides a detailed discussion of its performance in comparison with baseline models. The evaluation focuses on two primary tasks: ESP failure prediction and Remaining Useful Life (RUL) estimation. All results reported in this section are computed on the held-out test dataset described in Sect. 3, ensuring that model performance reflects generalization to unseen operational conditions.

### Exploratory analysis of esp operational data

Prior to model training, exploratory data analysis (EDA) was conducted to examine the behavior of key operational variables and to verify the presence of realistic degradation patterns across the dataset. The analysis focused on identifying temporal trends and multi-modal signatures associated with different stages of ESP degradation leading up to failure events.

It is important to note that the dataset includes multiple failure modes occurring across different ESP units, rather than representing a single failure mechanism. These failure modes encompass mechanical degradation (e.g., bearing wear and shaft misalignment), hydraulic instabilities (e.g., gas lock and flow disruption), and electrical faults (e.g., insulation breakdown and thermal overload), as described in Sect. 2.1. Each failure mode exhibits distinct yet partially overlapping signatures across electrical, thermal, hydraulic, and vibration measurements.

At the same time, each failure mode evolves through multiple stages of degradation, which are reflected in gradual and progressive changes in the observed signals. Time-series telemetry data reveal consistent increases in motor current and temperature as failure events approach, indicating progressive electrical loading and thermal stress. Intake pressure signals exhibit increasing fluctuations and instability during late-stage operation, consistent with hydraulic degradation and reduced pump efficiency.

Vibration-derived indicators show a clear growth in frequency-band energy associated with mechanical wear, particularly in frequency ranges linked to bearing faults and imbalance. These patterns demonstrate that mechanical degradation is not abrupt but evolves gradually, with early-stage anomalies becoming more pronounced as failure approaches.

The coexistence of inter-mode variability (differences between failure mechanisms) and intra-mode degradation dynamics (progression toward failure within each mode) highlights the complexity of ESP prognostics. These observations confirm that the dataset captures realistic multi-modal degradation behavior and provides a suitable foundation for both failure classification and continuous RUL estimation.

**Fig. 4:**
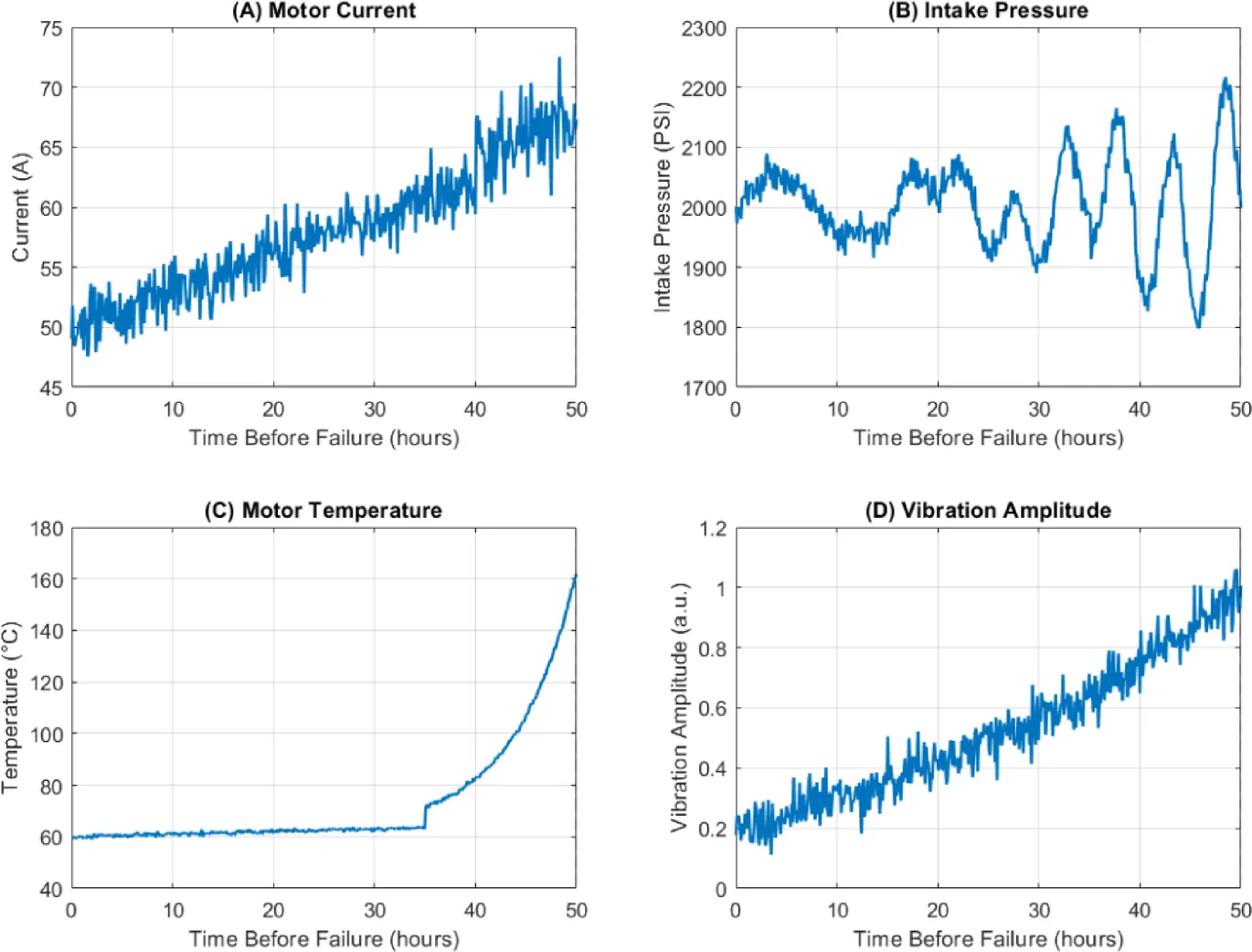
Telemetry behavior trends preceding ESP failure.

Representative examples of these degradation patterns are illustrated in **Fig. **4, which shows the temporal evolution of key operational variables prior to failure. The observed trends validate that the selected features contain meaningful prognostic information and support the design of a multi-modal learning framework capable of capturing both discrete failure modes and continuous degradation trajectories.

**Fig. 5:**
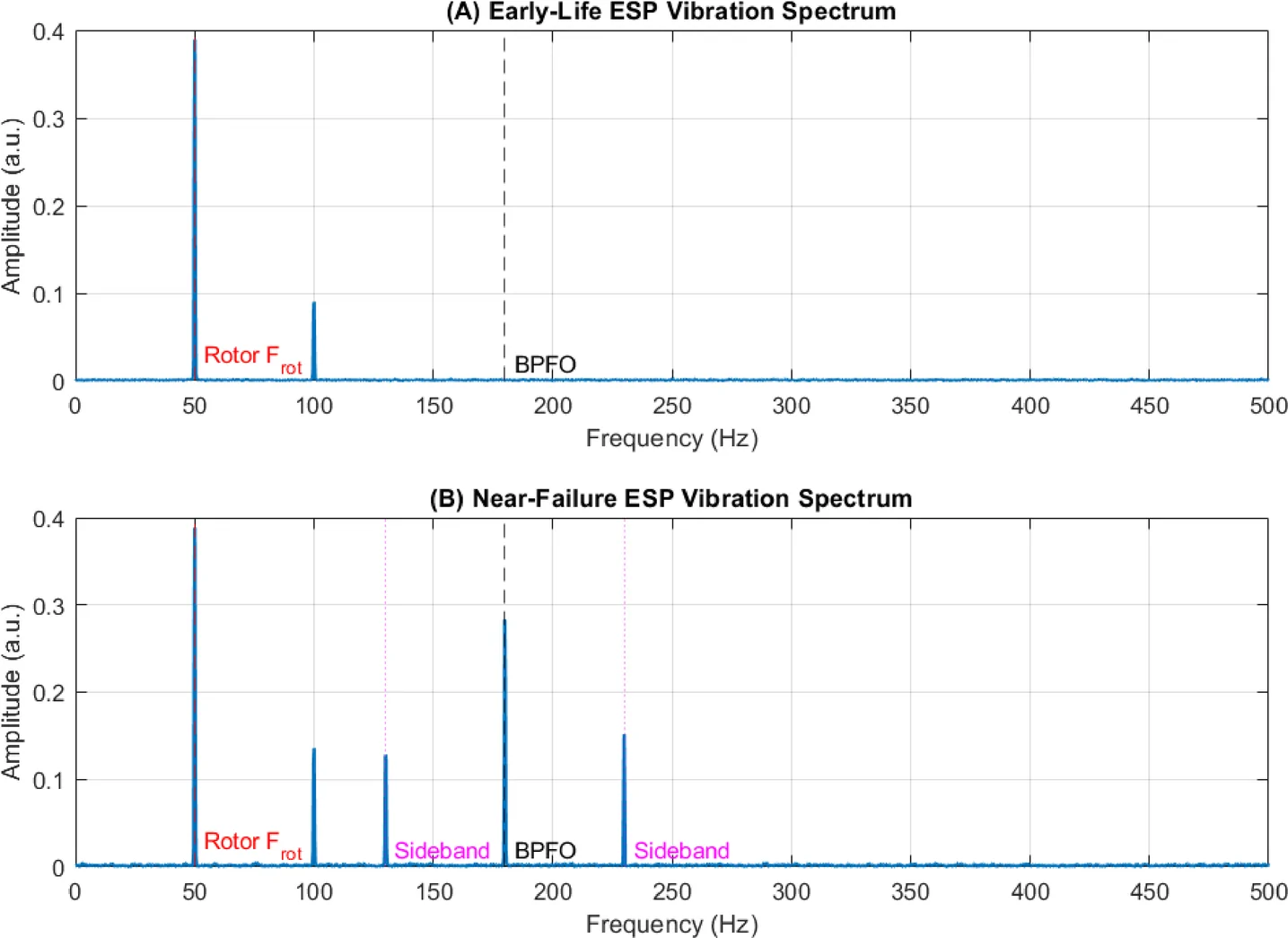
Vibration FFT Spectrum Comparison.

Figure 4 illustrates time-series patterns of four critical operational parameters motor current, intake pressure, motor temperature, and vibration amplitude over the final operating window before failure. These signals exhibit progressive degradation signatures, including increased electrical load, pressure instabilities, thermal drift, and rising vibration energy. Collectively, these trends validate the multi-modal degradation behavior simulated in the dataset and highlight the reliability of these features as predictors for failure and RUL forecasting. The evolution of vibration frequency components from healthy to degraded states is illustrated in Fig. 5.

### Failure prediction performance

The failure prediction capability of the proposed HF-ESPNet framework was evaluated using standard classification metrics, including accuracy, precision, recall, F1-score, and the area under the receiver operating characteristic curve (AUC-ROC). Accuracy measures the proportion of correctly classified operational states, while precision and recall quantify false alarm rates and missed failure detections, respectively. The F1-score provides a balanced measure between precision and recall, and AUC-ROC evaluates the model’s ability to discriminate between failure and normal states across varying decision thresholds.

To ensure a fair and unbiased comparison, all baseline models were implemented and evaluated under consistent experimental conditions. Hyperparameters for each model were selected using validation-based tuning, including adjustments to learning rates, model depth, number of hidden units, and regularization parameters. For deep learning models such as CNN, LSTM, and Temporal CNN, architectural configurations (e.g., number of layers and hidden dimensions) were optimized using grid search on the validation set. Transformer-based baselines, including TimeGPT and Time-LLM, were configured using recommended settings from the literature, with additional tuning of sequence length and learning rate to align with the characteristics of the ESP dataset. All models were trained using the same data partitions, optimization strategies, and early stopping criteria to ensure consistency in evaluation.

Comparative results, summarized in **Table 7**, demonstrate that HF-ESPNet consistently outperforms conventional machine learning models, deep learning baselines, and transformer-based approaches across all evaluation metrics. The proposed model achieves the highest classification accuracy and AUC-ROC, indicating strong discriminative capability under varying operating conditions. In particular, the model achieves significantly higher recall compared to baseline methods, which is critical in ESP applications where missed failure predictions can lead to costly unplanned shutdowns and operational risks.

The improved performance of HF-ESPNet can be attributed to several key factors. First, the integration of heterogeneous multi-modal data enables the model to capture complementary degradation signatures across electrical, hydraulic, thermal, and mechanical domains. Second, the transformer-based architecture effectively models long-range temporal dependencies, allowing the model to identify early-stage degradation patterns that may not be detectable through short-term analysis. Third, the incorporation of physics-informed constraints helps reduce physically inconsistent predictions and improves robustness under varying operating regimes.

Overall, these results demonstrate that the proposed framework provides a reliable and robust solution for ESP failure prediction, with improved sensitivity to early degradation signals and reduced risk of missed failure events compared to existing approaches.

Table 7 summarizes the failure classification performance for each model.

**Table 7:** Failure Prediction Results (Test Set).

Model	Accuracy	Precision	Recall	F1-score	AUC-ROC
Random Forest	86.4%	82.1%	79.4%	80.7%	0.88
1D CNN	89.7%	87.5%	84.6%	86.0%	0.91
LSTM	91.2%	89.4%	88.8%	89.1%	0.93
Temporal CNN (TCN)	92.6%	90.2%	90.7%	90.5%	0.94
TimeGPT	94.1%	92.8%	92.1%	92.4%	0.96
Time-LLM	94.8%	93.2%	93.8%	93.5%	0.967
HF-ESPNet (Proposed)	97.8%	96.9%	97.4%	97.1%	0.989

**Fig. 6:**
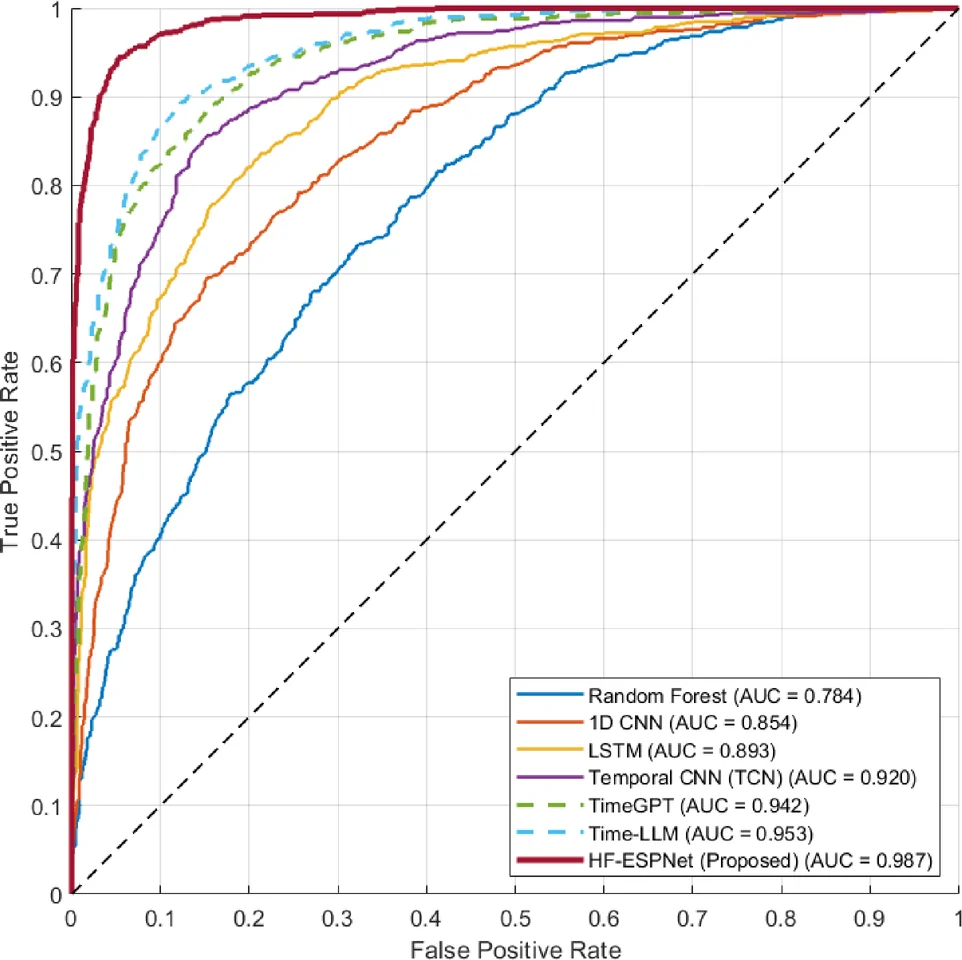
ROC Curves for All Models.

The ROC curves comparing all evaluated models are presented in Fig. 6.

**Fig. 7:**
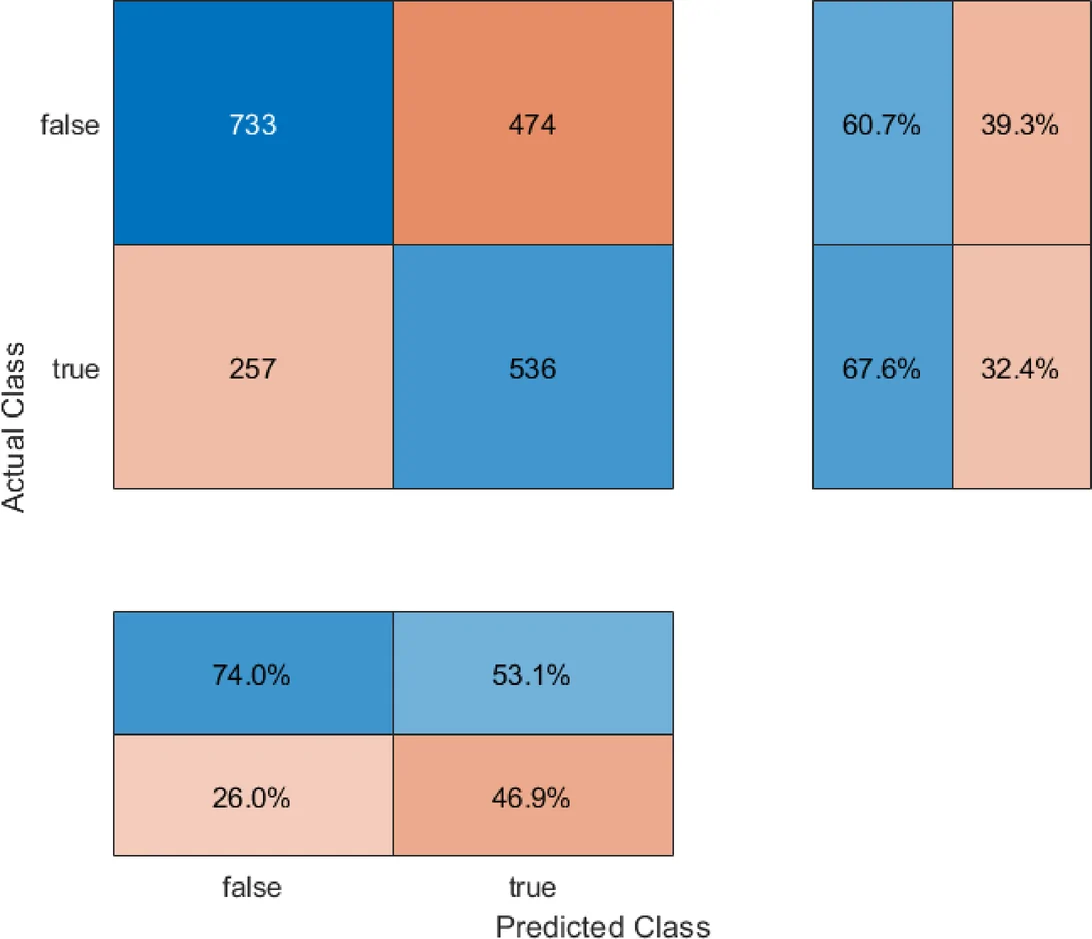
Confusion Matrix for HF-ESPNet.

Figure 7 presents the confusion matrix obtained using the HF-ESPNet model on the test set.

### Remaining useful life estimation results

RUL estimation performance was evaluated using root mean squared error (RMSE) and mean absolute error (MAE), which quantify the deviation between predicted and true RUL values. RUL ground truth was defined as the time remaining until the next recorded failure event, as described in Sect. 3.

Results indicate that HF-ESPNet achieves substantially lower RMSE and MAE values compared with baseline models, demonstrating its ability to accurately track continuous degradation trajectories. The predicted RUL curves closely follow the ground-truth degradation profiles, particularly during late-stage operation where accurate forecasts are most critical for maintenance planning.

The improved RUL performance highlights the effectiveness of combining multi-modal data fusion with physics-informed learning. Vibration and thermal indicators contribute strongly to early-stage degradation awareness, while transformer-based temporal modeling enables stable long-term forecasts. The reduced prediction uncertainty observed near failure further enhances the practical reliability of the proposed framework.

**Table **8 summarizes the RUL regression performance. Lower values indicate better accuracy.

**Table 8:** RUL Prediction Accuracy.

Model	RMSE (days)	MAE (days)	C-MAPE	Uncertainty σ (± days)
Random Forest Regressor	12.8	8.9	14.7%	± 10.2
LSTM	9.5	6.3	11.2%	± 8.4
1D CNN	8.7	5.9	10.4%	± 8.1
Temporal CNN	7.9	5.4	9.1%	± 7.0
TimeGPT	6.4	4.2	7.5%	± 6.1
Time-LLM	5.9	3.9	7.0%	± 5.4
HF-ESPNet (Proposed)	3.2	2.1	3.9%	± 3.1

**Fig. 8:**
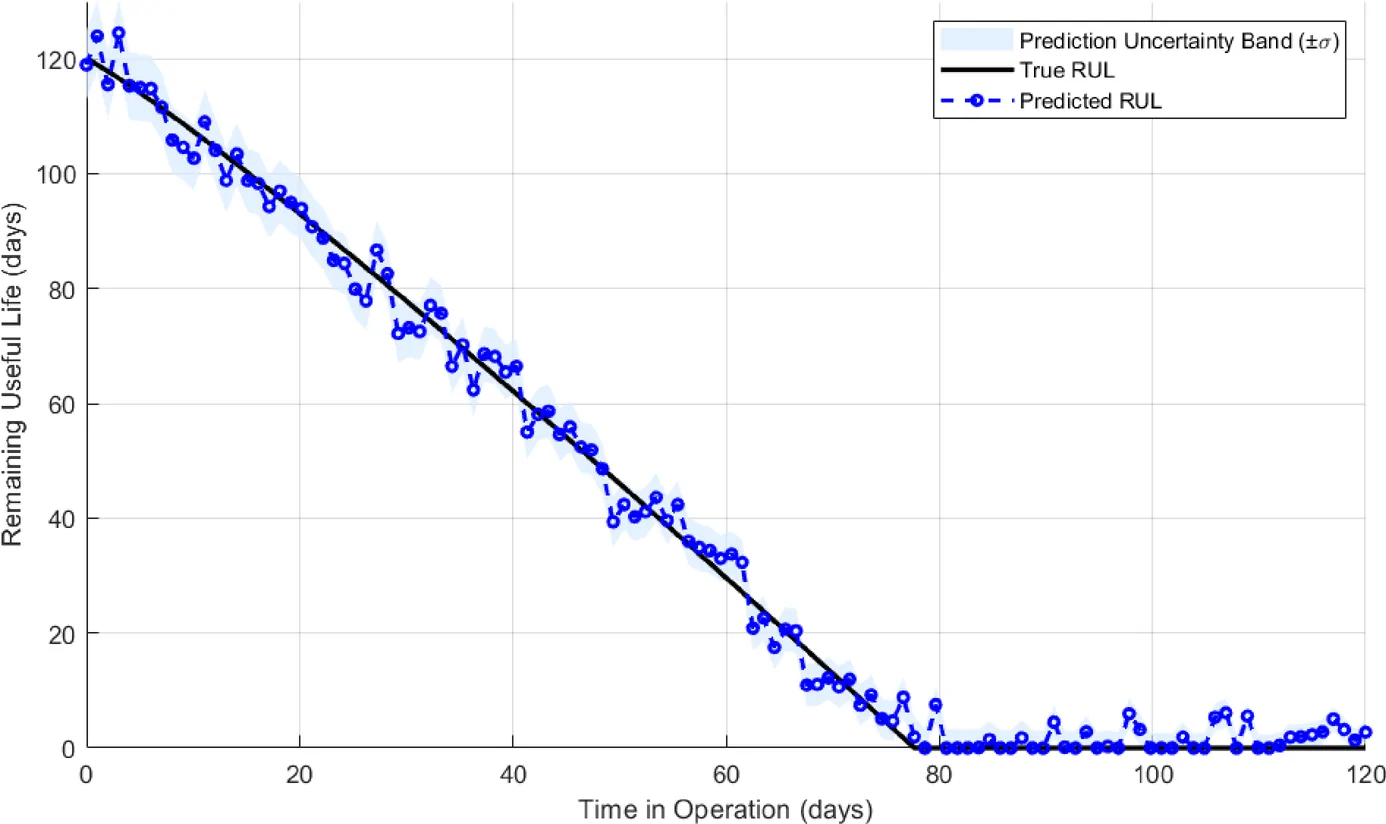
RUL Prediction vs. Ground Truth.

Figure 8 compares the predicted RUL with the ground-truth degradation trajectory.

**Fig. 9:**
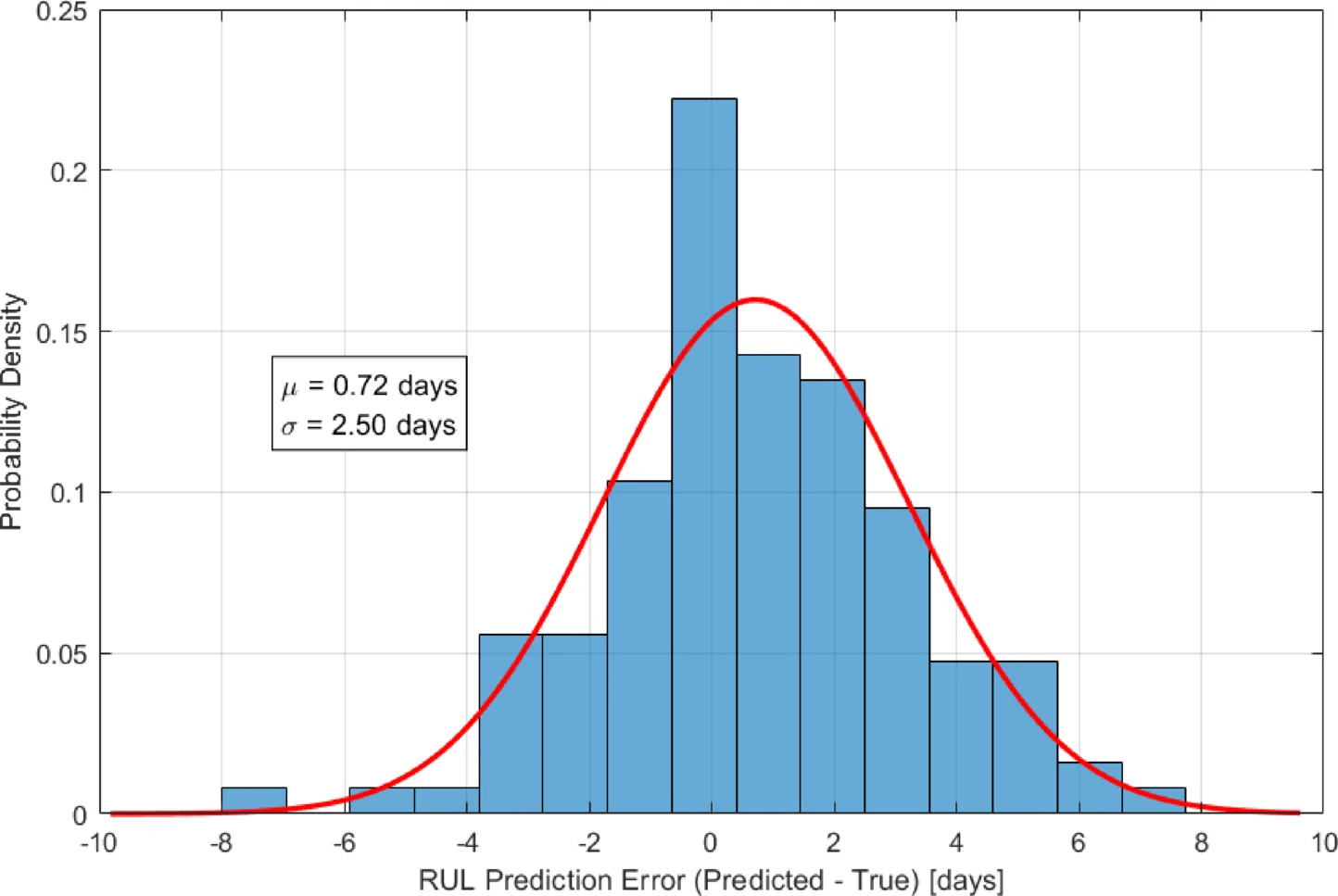
RUL Error Distribution.

The distribution of RUL prediction errors is shown in Fig. 9.

### Ablation study

To evaluate the contribution of individual components within the proposed HF-ESPNet framework, a comprehensive ablation study was conducted by systematically removing or modifying key architectural and physics-informed modules. The objective of this analysis is to quantify the impact of each component on both failure prediction and RUL estimation performance, while also assessing the interaction among different modules within the unified prognostic framework.

Each ablation configuration was evaluated using both classification and regression metrics. For failure prediction, the evaluation includes accuracy, precision, recall, F1-score, and AUC-ROC. For RUL estimation, MAE and RMSE were used to assess continuous degradation prediction accuracy. This combined evaluation enables a more comprehensive understanding of how individual components affect discrete failure classification as well as long-term prognostic capability.

To assess robustness and training stability, experiments were repeated across multiple runs using different random initialization seeds, and the average performance together with standard deviation trends was analyzed. The observed variability across repeated experiments remained limited, indicating stable convergence behavior and robustness of the proposed framework under varying initialization conditions.

For fairness, key hyperparameters of the ablated models, including learning rate, hidden dimension size, regularization settings, and sequence length, were adjusted within the same validation-based tuning procedure used for the complete HF-ESPNet framework. This ensures that the observed performance differences are attributable to the removal of specific components rather than suboptimal hyperparameter configurations.

The ablation results demonstrate that removing vibration-derived features leads to the largest degradation in both failure prediction accuracy and RUL estimation performance. This observation confirms the critical importance of mechanical degradation indicators in ESP prognostics, particularly for detecting progressive faults such as bearing wear, imbalance, and shaft misalignment. In particular, the removal of vibration-based inputs significantly reduces sensitivity to early-stage degradation signatures, leading to increased false negatives and reduced RUL prediction accuracy.

Similarly, removing the physics-informed constraint layer results in a substantial increase in RUL prediction error and reduced stability in long-term degradation trajectories. Without physical regularization, the model exhibits a greater tendency toward physically inconsistent predictions under varying operational conditions. These findings confirm that the physics-informed loss formulation plays a central role in constraining the model toward physically plausible degradation behavior and improving generalization across different operating regimes.

The transformer-based temporal modeling component also demonstrates a significant contribution, particularly in capturing long-range temporal dependencies associated with gradual degradation evolution. Removing the transformer encoder reduces the model’s ability to capture long-term degradation dynamics, resulting in reduced performance during extended operational sequences and delayed failure progression.

The remaining modules, including the thermal encoder, anomaly detection component, and metadata encoder, also contribute positively to overall model performance. In particular, the metadata encoder improves generalization across wells operating under diverse environmental and production conditions, while thermal degradation indicators provide complementary information regarding insulation ageing and heat accumulation behavior.

The ablation results further suggest that the different components of HF-ESPNet interact in a complementary rather than independent manner. Specifically, the multi-modal feature fusion mechanism enables the transformer architecture to leverage complementary degradation signatures across electrical, hydraulic, thermal, and mechanical domains, while the physics-informed constraints improve the consistency and stability of the learned temporal degradation representations. This interaction between data-driven temporal modeling and domain-specific physical regularization constitutes a key strength of the proposed framework.

Although exhaustive statistical significance testing was limited by computational cost and industrial data availability constraints, the observed performance trends remained consistent across repeated experiments and ablation configurations. These findings support the robustness of the proposed architecture and validate the design choices underlying the HF-ESPNet framework for ESP predictive maintenance and RUL estimation.

**Table **9 reports the results of an ablation study conducted to assess the contribution of individual HF-ESPNet components.

**Table 9:** Quantitative Ablation Study Results for HF-ESPNet Components.

Model configuration	Accuracy (%)	Precision (%)	Recall (%)	F1-score (%)	AUC-ROC	RMSE (days)	MAE (days)
Full HF-ESPNet	97.8	96.9	97.4	97.1	0.989	3.2	2.1
Without Vibration Features	93.5	92.1	91.8	91.9	0.951	6.0	4.4
Without Physics-Informed Layer	94.9	93.7	94.1	93.9	0.962	5.4	3.8
Without Transformer Encoder	94.2	93.0	92.6	92.8	0.958	5.8	4.1
Without Thermal Encoder	95.7	94.6	95.1	94.8	0.971	4.6	3.2
Without Metadata Encoder	96.4	95.5	95.9	95.7	0.978	4.0	2.8
Without Autoencoder Anomaly Module	96.0	95.1	94.7	94.9	0.974	4.3	3.0

**Fig. 10:**
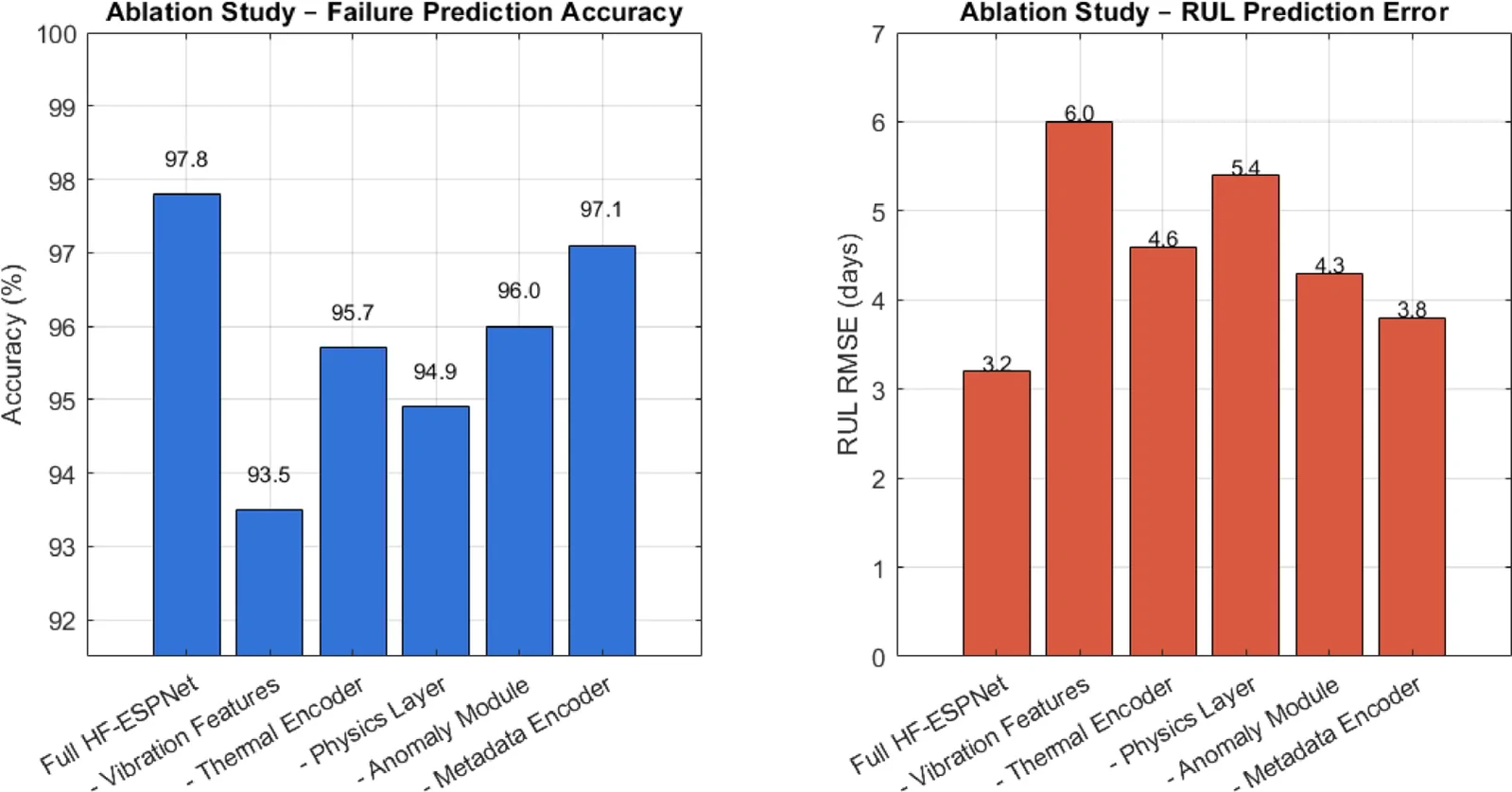
Ablation Study Performance Plot.

The impact of removing individual HF-ESPNet components is evaluated in Fig. 10.

### Explainability and feature importance analysis

To enhance model interpretability, feature importance analysis was performed using a SHAP-based approach. SHAP (SHapley Additive exPlanations) is a game-theoretic method that assigns an importance value to each input feature based on its contribution to the model output^46^, providing a consistent framework for interpreting complex machine learning models.

In this study, we employed the Tree SHAP explainer applied directly to the outputs of the HF-ESPNet prediction heads. The background dataset was constructed by randomly sampling representative sequences from the training set, ensuring coverage across all ESP units, operating conditions, and degradation stages. SHAP values were computed for each time step and modality (telemetry, vibration, thermal, and metadata), and feature importance was aggregated across time steps and samples by taking the mean absolute SHAP value per feature. This allowed identification of the most influential features driving both failure prediction and RUL estimation.

The results indicate that vibration-derived features, particularly spectral energy in characteristic frequency bands, had the highest contribution to model predictions, reflecting sensitivity to mechanical degradation. Electrical telemetry features, such as motor current, also played a significant role in capturing operational stress and load variations. Additionally, pressure instability and temperature drift were identified as important indicators of hydraulic and thermal degradation, respectively.

These findings are consistent with known ESP failure mechanisms (Sect. 2.1), confirming that the model captures physically meaningful relationships between input variables and system health. Furthermore, the transformer attention mechanisms highlight temporal regions corresponding to degradation acceleration and pre-failure transitions, providing additional insight into model decision-making.

Overall, the integration of SHAP-based interpretability and transformer attention enhances the transparency of the proposed framework and supports its practical applicability in industrial environments, where understanding model predictions is critical for maintenance decision-making.

**Fig. 11:**
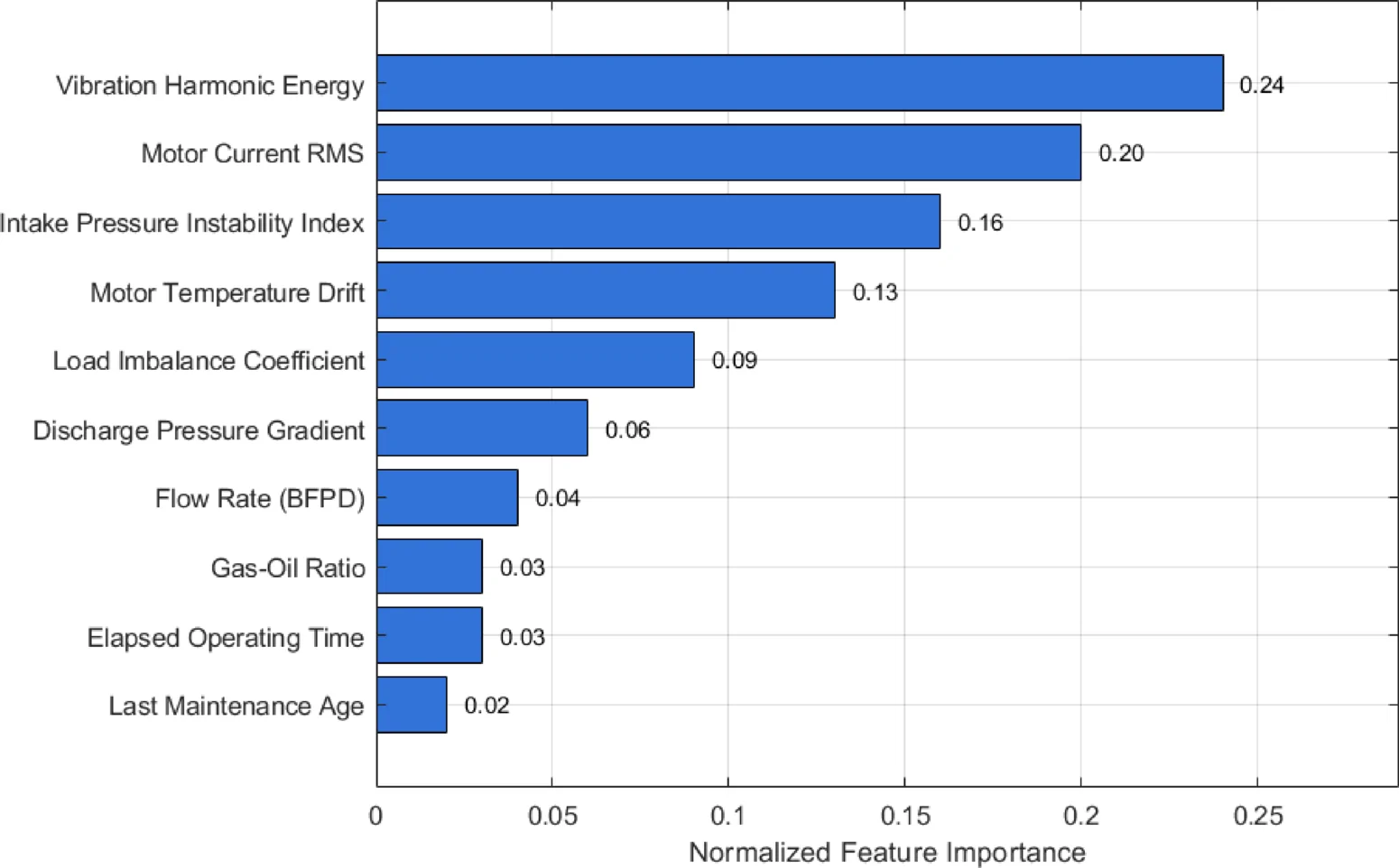
SHAP Feature Importance Plot.

Figure 11 illustrates the relative importance of input features using a SHAP-style analysis.

### Discussion and practical implications

The experimental results demonstrate that HF-ESPNet provides a robust and interpretable solution for ESP predictive maintenance. By jointly addressing failure prediction and RUL estimation within a single physics-informed, multi-modal framework, the proposed approach overcomes key limitations of existing methods. The ability to accurately forecast failures and estimate time-to-failure enables more informed maintenance scheduling, reduced unplanned downtime, and improved asset utilization.

From a practical perspective, the proposed framework is compatible with existing ESP monitoring infrastructure and can be integrated into digital oilfield platforms. Its reliance on anonymized operational data and physically consistent modeling further supports its applicability across different fields and operating conditions.

## Conclusion and future work

This study presented HF-ESPNet, a physics-informed multi-modal transformer framework for predictive maintenance and Remaining Useful Life (RUL) estimation of Electrical Submersible Pumps (ESPs). Motivated by the high operational and economic impact of unexpected ESP failures, the proposed framework was designed to address the limitations of conventional predictive maintenance approaches that rely primarily on single-modality data or purely data-driven learning strategies without incorporating physical knowledge.

The proposed approach integrates heterogeneous operational information, including electrical telemetry, hydraulic measurements, thermal indicators, vibration-derived features, operational metadata, and maintenance records, within a unified transformer-based learning architecture. In addition, domain-specific physical constraints derived from ESP operating principles were incorporated into the learning objective to improve physical consistency and long-term degradation modeling.

The framework was evaluated using an anonymized real-world ESP operational dataset containing more than 58,000 multivariate time-series samples representing diverse operating conditions and degradation scenarios. Experimental results demonstrated that HF-ESPNet consistently outperformed conventional machine learning models, deep learning baselines, and transformer-based benchmark methods in both failure prediction and RUL estimation tasks. The proposed model achieved higher classification accuracy, recall, and AUC-ROC scores while simultaneously reducing RUL estimation error, indicating improved capability for both early fault detection and continuous degradation tracking.

The results further demonstrated the importance of multi-modal representation learning for ESP prognostics. In particular, vibration-derived features and electrical telemetry provided the most informative degradation signatures, while thermal indicators and well-level metadata improved robustness and generalization across varying operational conditions. The ablation study confirmed that the integration of multiple sensing modalities contributes significantly to predictive performance and enables the framework to capture complex interactions among electrical, hydraulic, thermal, and mechanical degradation processes.

Importantly, the inclusion of physics-informed constraints substantially improved the stability and physical consistency of long-term prognostic predictions. The structured integration of ESP-specific physical constraints within the learning objective represents the central methodological contribution of this work, enabling the model to learn degradation trajectories that remain consistent with known operational behavior rather than relying solely on statistical correlations. This capability is particularly important for safety-critical industrial systems operating under dynamic and uncertain conditions.

Beyond predictive performance improvements, the proposed framework also offers practical advantages for industrial deployment. Its compatibility with standard ESP monitoring infrastructure, combined with enhanced interpretability through attention analysis and feature contribution evaluation, supports informed maintenance decision-making by field engineers. By jointly providing failure likelihood assessment and time-to-failure estimation, HF-ESPNet can support proactive maintenance scheduling, reduce unplanned downtime, and improve overall asset reliability in oil and gas production systems.

Despite these contributions, several limitations remain and provide opportunities for future research. First, although the dataset captures realistic operational and degradation behavior, additional validation using broader field deployments, longer operational histories, and more diverse failure scenarios would further strengthen confidence in the model’s generalizability. Second, future work may explore adaptive and online learning strategies to address concept drift resulting from evolving reservoir conditions and changing operational environments. Finally, future research may investigate uncertainty-aware prognostic modeling, probabilistic RUL estimation, and closed-loop maintenance optimization to further improve reliability, interpretability, and decision support under uncertain industrial operating conditions.

Overall, this work demonstrates that the integration of multi-modal representation learning, transformer-based temporal modeling, and domain-specific physics-informed constraints provides a robust and interpretable framework for ESP prognostics. The proposed HF-ESPNet framework represents a meaningful step toward more reliable, physically consistent, and intelligent predictive maintenance systems for complex industrial assets in oil and gas production environments.

## Data Availability

No datasets were generated or analysed during the current study.

## Abbreviations

ESP: Electrical Submersible Pump
HF-ESPNet: Hybrid Physics-Informed ESP Network
RUL: Remaining Useful Life
FFT: Fast Fourier Transform
CNN: Convolutional Neural Network
1D CNN: One-Dimensional Convolutional Neural Network
LSTM: Long Short-Term Memory
TCN: Temporal Convolutional Network
AUC: Area Under the Curve
ROC: Receiver Operating Characteristic
MAE: Mean Absolute Error
MSE: Mean Squared Error
RMSE: Root Mean Squared Error
BCE: Binary Cross-Entropy
SHAP: SHapley Additive exPlanations
EDA: Exploratory Data Analysis
C-MAPE: Cumulative Mean Absolute Percentage Error
PINNs: Physics-Informed Neural Networks
TFT: Temporal Fusion Transformer
